# Treatment with novel topoisomerase inhibitors in Ewing sarcoma models reveals heterogeneity of tumor response

**DOI:** 10.3389/fcell.2024.1462840

**Published:** 2024-10-24

**Authors:** Unsun Lee, Ludmila Szabova, Victor J. Collins, Melanie Gordon, Kristine Johnson, Deborah Householder, Stephanie Jorgensen, Lucy Lu, Laura Bassel, Fathi Elloumi, Cody J. Peer, Ariana E. Nelson, Sophia Varriano, Sudhir Varma, Ryan D. Roberts, Zoe Weaver Ohler, William D. Figg, Shyam K. Sharan, Yves Pommier, Christine M. Heske

**Affiliations:** ^1^ Pediatric Oncology Branch, Center for Cancer Research, National Cancer Institute, National Institutes of Health, Bethesda, MD, United States; ^2^ Center for Advanced Preclinical Research, Frederick National Laboratory for Cancer Research, National Cancer Institute, National Institutes of Health, Frederick, MD, United States; ^3^ Center for Advanced Preclinical Research, Center for Cancer Research, National Cancer Institute, National Institutes of Health, Frederick, MD, United States; ^4^ Developmental Therapeutics Branch, National Cancer Institute, National Institutes of Health, Bethesda, MD, United States; ^5^ Clinical Pharmacology Program, Center for Cancer Research, National Cancer Institute, National Institutes of Health, Bethesda, MD, United States; ^6^ Center for Childhood Cancer Research, Abigail Wexner Research Institute, Nationwide Children’s Hospital, Columbus, OH, United States; ^7^ Department of Hematology, Oncology and Bone Marrow Transplant, Nationwide Children’s Hospital, Columbus, OH, United States

**Keywords:** topoisomerase 1 inhibitor, indenoisoquinoline, indotecan, Ewing sarcoma, patient-derived xenograft, Ewing Sarcoma MinerCDB

## Abstract

**Introduction:**

The topoisomerase 1 (TOP1) inhibitor irinotecan is a standard-of-care agent for relapsed Ewing sarcoma (EWS), but its efficacy is limited by chemical instability, rapid clearance and reversibility, and dose-limiting toxicities, such as diarrhea. Indenoisoquinolines (IIQs) represent a new class of clinical TOP1 inhibitors designed to address these limitations.

**Methods:**

In this study, we evaluated the preclinical efficacy of three IIQs (LMP400, LMP744, and LMP776) in relevant models of EWS. We characterized the pharmacokinetics of IIQs in orthotopic xenograft models of EWS, optimized the dosing regimen through tolerability studies, and tested the efficacy of IIQs in a panel of six molecularly heterogeneous EWS patient-derived xenograft (PDX) models. For each PDX, we conducted whole genome and RNA sequencing, and methylation analysis.

**Results:**

We show that IIQs potently inhibit the proliferation of EWS cells in vitro, inducing complete cell growth inhibition at nanomolar concentrations via induction of DNA damage and apoptotic cell death. LMP400 treatment induced ≥30% tumor regression in two of six PDX models, with more durable regression compared to irinotecan treatment in one of these models. RNA sequencing of PDX models identified a candidate predictive biomarker gene signature for LMP400 response. These data, along with pharmacogenomic data on IIQs in sarcoma cell lines, are available at a new interactive public website: https://discover.nci.nih.gov/rsconnect/EwingSarcomaMinerCDB/.

**Discussion:**

Our findings suggest that IIQs may be promising new agents for a subset of EWS patients.

## Introduction

Ewing sarcoma (EWS) is an aggressive pediatric cancer and the second most common malignant bone tumor in children (Doyle, 2014). The defining molecular characteristic of EWS is a chromosomal translocation of the EWS RNA Binding Protein 1 (*EWSR1)* gene with an Erythroblast Transformation Specific (*ETS)* proto-oncogene (*FLI, ERG, ETV1, ETV4,* or *FEV*), which produces a chimeric protein that binds to DNA and induces downstream epigenetic dysregulation (Delattre et al., 1992). While chemotherapy has greatly improved outcomes for patients with localized EWS, effective treatments for patients with metastatic and relapsed EWS remains elusive, with 5-year overall survival rates of 30% and <15%, respectively (Balamuth and Womer, 2010; Gaspar et al., 2015; Stahl et al., 2011). Prognostic risk stratification for patients is still largely based on clinical features, though there are ongoing efforts to use molecular biomarkers, such as *STAG2* loss, *TP53* mutation, and copy number changes, to improve this process (Shulman et al., 2022).

Clinically, EWS tumors are highly sensitive to radiation therapy and DNA damaging agents, such as doxorubicin, cyclophosphamide, and etoposide, all of which are typically used as first-line therapy (Nesbit et al., 1990; Brown et al., 1987). For patients who have relapsed, irinotecan is a key component of first-line salvage therapy, where it has shown meaningful antitumor activity (Li et al., 2006; Raciborska et al., 2013; Casey et al., 2009; Wagner et al., 2007). Irinotecan is a camptothecin prodrug analog that selectively inhibits topoisomerase 1 (TOP1) and traps the DNA-TOP1 cleavage complex (TOP1cc), resulting in broken replication forks and impaired relaxation of supercoiled DNA (Li et al., 2006). However, numerous challenges, such as systemic toxicity and poor bioavailability of the active metabolite (SN-38), limit the clinical activity of irinotecan (Brangi et al., 1999; Hecht, 1998).

Indenoisoquinolines (IIQs) are a more recently developed class of non-camptothecin agents that selectively trap TOP1cc and possess several potential advantages over irinotecan (Thomas and Pommier, 2019). These include improved chemical stability, prolonged targeting of the TOP1cc, evasion of MDR efflux pumps, and absence of drug-induced diarrhea (Thomas and Pommier, 2019; Antony et al., 2007; Tanizawa et al., 1994; Burton et al., 2018; Covey et al., 1989). The IIQs LMP400 (indotecan), LMP776 (indimitecan), and LMP744 (MJ-III-65) have recently been evaluated in early phase clinical trials for adults with relapsed solid tumors and lymphomas but have yet to be tested in any pediatric indication (Burton et al., 2018; Kummar et al., 2016). Given the clinical activity of irinotecan in EWS, and the need for improved therapies for this malignancy, the purpose of our study was to evaluate the activity of IIQs LMP400, LMP744, and LMP776 in a diverse panel of EWS models. Here, we characterize the pharmacokinetics (PK), toxicity, and efficacy of IIQs in a diverse panel of preclinical cell line and patient-derived xenograft (PDX) models of EWS. We focused on LMP400 and compared it to the standard agent irinotecan, observing heterogeneity in responses across both agents with some models more responsive to irinotecan and others to LMP400. We have examined the expression profile of responders and non-responders to identify potential predictive biomarkers. Our findings suggest that LMP400 may be more effective than irinotecan in a subset of EWS.

## Materials and methods

Key reagents, including the compounds, antibodies, chemicals, and commercial assays, are listed in Supplementary Table S1. All assays described were performed following the manufacturer’s manual.

### Cell lines

EWS cell lines were authenticated by short tandem repeat DNA fingerprinting and confirmed to be mycoplasma negative. EW8, TC71, TC32, RDES, and 5838 have been previously described (Yeung et al., 2019). ES1, ES4, and ES6 were a gift from Dr. Peter Houghton (University of Texas Health Science Center, San Antonio, TX). All cell lines were maintained in RPMI growth medium (Life Technologies, Grand Island, NY) supplemented with 10% FBS (Millipore Sigma, St. Louis, MO), 100 U/mL penicillin and 100 ug/mL streptomycin (Thermo Fisher Scientific), and 2 mM L-glutamine (Thermo Fisher Scientific) at 37°C in 5% CO_2_.

### Compounds

IIQs LMP400 (indotecan, NSC 724998), LMP744 (MJ-III-65, NSC 706744), and LMP776 (indimitecan, NSC 725776) were provided by the Developmental Therapeutics Program (DTP), Center for Cancer Research (CCR), National Cancer Institute (NCI), National Institutes of Health (NIH, Bethesda, MD). Irinotecan was obtained from the NIH Veterinary Pharmacy (Bethesda, MD).

### Cell proliferation assays

The IncuCyte live-cell analysis system (Essen BioScience, Ann Arbor, MI) was used to monitor the real-time cellular proliferation of EWS cells. Cells were plated at a density of 2,000–4,000 cells/well in 96-well plates (at least 5 wells/condition), treated the following day, and followed longitudinally for proliferation. Each experiment was performed at least two times.

### Protein analysis

EWS cells were plated at 1 million cells/10-cm plate overnight, then treated and collected at different time points (24, 48, or 72 h) before lysing in 1X RIPA (Thermo Fisher Scientific) supplemented with phosphatase and protease inhibitor cocktail (Thermo Fisher Scientific). Protein was quantified by BCA protein assay (Thermo Fisher Scientific), and 30 µg of protein was separated on 4%-12% SDS-PAGE gels (Thermo Fisher Scientific) and transferred onto nitrocellulose membranes (Thermo Fisher Scientific). Membranes were blocked with 5% nonfat dry milk in TBS (20 mM Tris-HCl, pH 7.4) (KPL, Gaithersburg, MD), plus 0.1% Tween20 (MilliporeSigma, Burlington, MA). Membranes were incubated overnight with primary antibodies (Supplementary Table S1 **)**. Bands were visualized on a BioRad Image Lab camera using West Femto or Pico ECL detection reagent (Thermo Fisher Scientific).

For protein analysis of xenograft tumor tissue, approximately 50 mg of flash frozen tumor was homogenized in 1 mL of tissue protein extraction reagent (T-PER) (Thermo Fisher Scientific) supplemented with phosphatase and protease inhibitor cocktail (Thermo Fisher Scientific) using the TissueRuptor II system (Qiagen, Hilden, Germany). Protein lysates were handled as described above and incubated with primary antibodies (Supplementary Table S1 **)**.

### Comet assay

EWS cells were plated at 1 million cells/10-cm plate overnight, treated, and collected 24 h post-treatment using 0.05% trypsin (Thermo Fisher Scientific). Cells were counted and resuspended at 1 million cells/mL in cold PBS (Thermo Fisher Scientific), and analyzed using the CometAssay Single Cell Electrophoresis Kit (Bio-Techne, Minneapolis, MN) following the manufacturer’s instructions for the Alkaline Comet assay protocol. Samples were viewed using a Nikon Eclipse TE300 microscope and analyzed using the OpenComet software tool for tail DNA percent comparison. Each experiment was performed at least two times.

### Cell cycle analysis

EWS cells were plated at 1 million cells/10-cm plate overnight, treated, and collected at either 24, 48, or 72 h. Cells were washed with cold PBS and fixed with cold 70% ethanol/PBS overnight at −20 °C. Fixed cells were centrifuged at 200 × *g* for 10 min at 4°C, washed in cold PBS, resuspended in propidium iodide (PI)/Triton X-100 staining solution (0.1% Triton X-100 (Millipore Sigma) in PBS, DNAse-free RNAse A (Millipore Sigma), PI (Thermo Fisher Scientific)), then incubated at 37°C for 15 min and strained (Thermo Fisher Scientific). Samples were quantified using an LSRFortessa flow cytometer (BD Biosciences, San Jose, CA) and analyzed with FlowJo software (Vancouver, Canada). Each experiment was performed at least two times.

### Annexin V assay

EWS cells were plated at 1 million cells/10-cm plate overnight, treated, and collected at either 24, 48, or 72 h. Cells were washed with cold PBS and stained using annexin V-FITC apoptosis detection kit (Cayman Chemical, Ann Arbor, MI) according to the manufacturer’s instructions. Annexin V-FITC labeled apoptotic cells were quantified using an LSRFortessa flow cytometer (BD Biosciences) and analyzed with FlowJo software. Each experiment was performed at least two times.

## Statistical analysis

Statistical significance between two groups was determined using the Mann-Whitney test. *p* < 0.05 was considered significant.

### Animal studies

NCI-Frederick is accredited by AAALAC International and follows the Public Health Service Policy for the Care and Use of Laboratory Animals. Animal care was provided in accordance with the procedures outlined in the “Guide for Care and Use of Laboratory Animals” (National Research Council; 1996; National Academy Press; Washington, D.C.). All study protocols were approved by the NCI at Frederick Animal Care and Use Committee (Frederick, MD). Studies used 8–16 weeks-old NSG female mice (NOD.Cg-Prkdc scid II2rg tmlWil/SzJ) from Frederick National Laboratories (Frederick, MD). For cell line xenograft studies, TC32 cells were resuspended in Hank’s balanced salt solution (HBSS); for PDX studies, dissociated tumor cells were resuspended in Matrigel/HBSS at a 1:1 ratio. Two million cells (in 50 uL) were injected into the periosteal region of the tibia of NSG mice. LMP400 and LMP744 were dissolved in one part 20 mM HCl/10 mM citric acid and nine parts 5% dextrose water; LMP776 was dissolved in one part 10 mM citric acid and nine parts 5% dextrose water. Vehicle groups received a solution containing one part 20 mM HCl/10 mM citric acid and nine parts 5% dextrose water. Irinotecan (20 mg/mL stock solution) was diluted with saline before use. For all studies, mice were weighed twice weekly. For studies utilizing tumor-bearing mice, tumor growth was monitored by caliper measurement twice weekly, and tumor volume was calculated as follows: (L × W^2^)/2, where L and W represent tumor length and width, respectively.

### Patient-derived xenografts (PDXs)

XEN-EWS-021 was generated in the Pediatric Oncology Branch (Center for Cancer Research (CCR), National Cancer Institute (NCI), National Institute of Health (NIH)) and has been previously described (Heske et al., 2016). SJEWS-18-09520, SJEWS049193_X1, and SJEWS-17-06841were obtained from the St. Jude Childhood Solid Tumor Network (Stewart et al., 2017). NCH-EWS-1 and NCH-EWS-4 were generated by Dr. Ryan Roberts (Nationwide Children’s Hospital, Columbus, OH). Confirmation of molecular signature was performed on each PDX, and cells were tested for mouse pathogens prior to injection. Following initial implantation, engraftment, and tumor growth to 1.7 cm in any direction, the p+1 passage was established, and tissue was harvested for downstream analyses and viable banking. Subsequent PDX passages were generated from p+1 viably banked cells. Histopathological examination of tumors from established PDXs and immunohistochemistry staining for CD99 confirmed EWS histology in all samples (Supplementary Figure S1). For additional details of histopathological analysis, see Supplementary Data Sheet S1.

### Pharmacokinetic (PK) analysis

Mice bearing TC32 tumors (600–1,200 mm^3^ volume) were dosed intravenously (IV) or intraperitoneally (IP) with a single dose of LMP400 at 10 mg/kg, and plasma and tumor samples were collected at 5 min, 30 min, 1, 2, 8, and 24 h later. A separate cohort of TC32 tumor-bearing mice were administered a single dose of LMP744 at 20 mg/kg or LMP776 at 10 mg/kg IV or IP, and plasma and tumor tissue were collected 5 min and 2 h later. Control samples were collected from vehicle-treated (IV or IP) mice 2 h post-dose. Bioanalysis of plasma and tissue concentrations of IIQs were performed using validated LC-MS assays, described in detail in Supplementary Data Sheet S1. PK parameters were calculated using noncompartmental methods using a naïve-pooled approach for destructive sampling. For AUC, a linear up-log down trapezoidal rule was used. At least three terminal points with measurable drug above the assay limit of quantification were used for the estimate of the elimination rate (slope of line r^2^ > 0.8). PK analysis was performed using Phoenix WinNonlin v8.3 (Certara Corp, Princeton, NJ).

### Tolerability studies

Tolerability studies of the IIQs were conducted in non-tumor bearing NSG mice. The doses and treatment schedules tested are shown in Supplementary Table S2, S3.

### Efficacy studies

Mice bearing PDX tumors were enrolled into efficacy studies when tumor volumes reached 300-1,000 mm^3^. All IIQ treatments were administered IV at 10 mg/kg following the 5-day-on/9-day-off/5-day-on treatment regimen. Irinotecan was administered IP at 2.5 mg/kg on the same schedule. Pilot efficacy studies comparing the efficacy of the IIQs versus vehicle were conducted using models SJEWS-17-06841 and SJEW-18-09520 (n = 3 mice/condition). Expanded efficacy studies comparing the efficacy of LMP400 versus vehicle versus irinotecan were conducted in six PDX models (XEN-EWS-021, SJEWS049193_X1, SJEW-18-09520, SJEWS-17-06841, NCH-EWS-1, and NCH-EWS-4) (n = at least 10 mice/condition).

### *In Vivo* statistical analysis

Log-rank (Mantel-Cox) test was performed to compare survival between groups with *p* < 0.05 considered significant.

### Whole genome sequencing

DNA isolated was from untreated flash frozen samples collected from p+1 PDX tumors. For each model, three biological replicates from different mice were collected. DNA extraction was performed simultaneously with the total RNA extraction using the AllPrep DNA/RNA Micro Kit (Qiagen) according to the manufacturer’s directions. The library was sequenced on Illumina NovaSeq 6000 S4 run using TruSeq Nano DNA prep and paired-end sequencing mode. The samples had 889M–1403M pass filter reads, with Q30 above 88%. The samples were mapped, and variants were called using DRAGEN.

Percent total mapping against reference genome hg38 was about 93%, and uniquely mapped reads were above 70%. Library complexity (i.e., percentage of non-duplicate reads) was determined by measuring the percentage of unique fragments in the mapped reads using MarkDuplicate utility. Percent duplicated reads were between 7% and 9%. Coverage statistics were also measured using DRAGEN. The mapped sequencing depth coverage (after alignment and marking duplicates) was between 32X and 57X. The mean insert size for these samples was between 550 and 614 bases. More than 84% of the genome had coverage above 20X.

We annotated the mutations using Annovar (Wang et al., 2010) to find the location of the mutation with respect to the gene, the putative effect of the mutation on protein function (whether deleterious or not using SIFT and Polyphen2) (Ng and Henikoff, 2003; Adzhubei et al., 2010), and its presence in the ExAC normal variation database (Karczewski et al., 2017). We selected mutations with at minimum read depth of 6 and a quality score>60 (if insertion or deletion) or >30 (if point mutation). Then we selected mutations that are putatively somatic (ExAC frequency of occurrence of 0) and deleterious (SIFT score <0.05 or a Polyphen2 score ≥ 0.85). For each gene we combined the mutation Variant Allele Frequencies (VAFs) to one score, using the “Genetic variant summation” method previously published in (Reinhold et al., 2014).

### RNA-sequencing

Total RNA was isolated from untreated flash frozen samples collected from p+1 PDX tumors. For each model, three biological replicates from different mice were collected. Total RNA extraction was performed using the AllPrep DNA/RNA Micro Kit (Qiagen) according to the manufacturer’s directions. DNase digestion was carried out using the RNase-Free DNase Set (Qiagen). The quality and quantity of extracted RNA samples were assessed by Nanodrop and Agilent4150 & 4200 TapeStation Systems at the CCR Genomics Core (Bethesda, MD). All samples were confirmed to have an RNA integrity number greater than 9.0 and were processed for library construction and sequencing at the CCR Sequencing Facility at the Frederick National Laboratory. Samples were sequenced on an Illumina NovaSeq 6000 S1 using Illumina Stranded Total RNA Prep, Ligation with Ribo-Zero Plus, and paired-end sequencing with a read length of 100 base pair (bp).

Reads of the samples were trimmed for adapters and low-quality bases using Cutadapt (Martin, 2011) before alignment with the reference human genome (hg38) and the annotated transcripts using STAR (Dobin et al., 2013). The average mapping rate of all samples was 86%. Unique alignment was above 56%. There were 5.56%–28.72% unmapped reads. The mapping statistics were calculated using Picard software. The samples had 0.02% ribosomal bases. Percent coding bases were between 27% and 47%. Percent UTR bases were 26%–34%, and mRNA bases were between 53% and 81% for all the samples. Library complexity was measured in terms of unique fragments in the mapped reads using Picard’s MarkDuplicate utility. The samples had 57%–72% non-duplicate reads. Gene expression quantification analysis was performed for all samples using STAR/RSEM tools (Li and Dewey, 2011).

Gene expression analysis using the raw counts comparing the non-responder and responder EWS PDXs was performed with limma + voom in the limma package (Law et al., 2014). The volcano plot was generated using R software and log_2_FC data. LASSO (least absolute shrinkage and selection operator) feature selection algorithm (Luna et al., 2021), was used to look for predictor genes for LMP400 treatment response. We identified 15 genes, from which 10 were considered significant (limma-voom calculated |fold change|>2.5 and *p*-value<0.05) and homogeneously expressed within each group (non-responder versus responder). Three additional genes, *CDKN1A*, *SIRT1*, and *FGFR2* were identified utilizing LASSO within the DNA replication and oncogene gene sets; they were also considered significant and homogeneously expressed within response groups.

Fusion data were generated using the DRAGEN fusion pipeline for each of the 18 PDX samples. We present the fusion results for *EWSR1-FLI1* in Supplementary Dataset S2.

### Methylation and copy number analysis

Raw PDX data (idat files) were processed using SeSame package (Lee et al., 2024) based on the hg19 reference genome. Each probe on the array measures the methylation status of one CpG site, i.e., it computes the ratio of the intensity of the methylated probe to the methylated plus unmethylated probe intensities (a value between 0-unmethylated to 1-methylated). For each gene, we selected a set of probes based on promoter and body regions and computed the average methylation value for all of the selected probes per region to calculate the promoter and body gene level values. Details on probe selection can be found at the following references (Reinhold et al., 2017; Pongor et al., 2022). The Bioconductor ChAMP package (Tian et al., 2017) was used to compute the log2 copy number at each CpG site. The average for all the CpG sites mapped to a gene is represented as the average log copy number of that gene.

### Integration of PDX data into the Ewing Sarcoma MinerCDB tool

Genomic data for the biological triplicates of the six PDXs were uploaded to the new Ewing Sarcoma MinerCDB site (https://discover.nci.nih.gov/rsconnect/EwingSarcomaMinerCDB/) following the architecture of our recently published Sarcoma_CellminerCDB website (Tlemsani et al., 2024). These data include transcriptome, methylome, copy number, and mutations for all genes, as well as drug response data for LMP400 and irinotecan for each of the individual PDX replicates. Drug responses are presented as response (>30% tumor reduction) designated as 1 or non-response (tumor reduction <30%) designated as 0.

### Availability of data

The data included in this study are available within the supplemental data files, and at the Ewing Sarcoma MinerCDB site: https://discover.nci.nih.gov/rsconnect/EwingSarcomaMinerCDB/. Additional data are available upon request from the corresponding author.

## Results

### Indenoisoquinolines (IIQs) induce DNA damage and apoptotic cell death in a diverse panel of EWS cell lines

**FIGURE 1:**
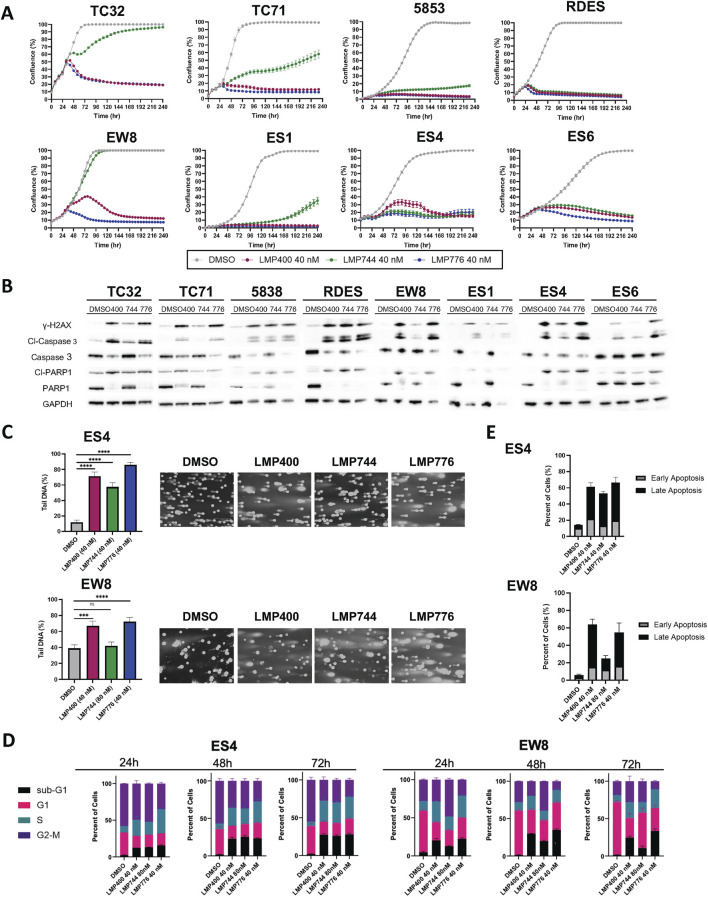
Indenoisoquinolines (IIQs) induce DNA damage and apoptotic cell death in EWS cell lines. **(A)**. IncuCyte live-cell analysis of eight EWS cell lines (TC32, TC71, 5838, RDES, EW8, ES1, ES4, and ES6) treated with either DMSO (grey), LMP400 (magenta), LMP744 (green), or LMP776 (blue) at 40 nM after overnight plating. **(B)**. Western blot analysis of total and cleaved PARP1, total and cleaved Caspase-3, and phosphorylated H2AX protein expression in EWS cell lines following 48 h of treatment with DMSO, LMP400, LMP744, or LMP776 at 40 nM. GAPDH was used as a loading control. **(C)**. Comet assay of EW8 and ES4 cell lines treated for 24 h with DMSO, LMP400 (40 nM), LMP744 (80 nM for EW8 and 40 nM for ES4), or LMP776 (40 nM). Mann-Whitney test was performed for each experiment (**** indicates *p* ≤ 0.0001, *** indicates *p* ≤ 0.001). **(D)**. Cell-cycle analysis of EW8 and ES4 cell lines treated for 24, 48, and 72 h with DMSO, LMP400, LMP744, and LMP776 using the same concentrations as in panel **(C)**. **(E)**. Apoptosis assay depicting Annexin V and PI staining of EW8 and ES4 cell lines treated for 72 h with the same concentrations listed in panels **(C)** and **(D)**.

To assess the *in vitro* activity of IIQs in EWS, we selected a panel of eight EWS cell lines (EW8, TC71, TC32, RDES, 5838, ES1, ES4, and ES6), reflecting a broad diversity of clinically relevant molecular features, including models with wild-type and mutated TP53 and STAG2 (Supplementary Table S4). Cells were treated with either LMP400, LMP744, or LMP776 at a range of doses, and longitudinal proliferation was analyzed using IncuCyte live-cell analysis. Across the cell line panel, each IIQ resulted in a loss of proliferation, with LMP776 the most potent and LMP744 the least potent of the IIQs. In all cell lines, treatment with LMP400 or LMP776 at 40 nM resulted in complete growth inhibition, while LMP744 achieved a complete inhibitory effect at 80 nM (Figure 1A; Supplementary Figure S2).

We next sought to assess the effects of IIQs on DNA integrity and to characterize the mechanism of cell growth inhibition. Immunoblot analyses of cells following 48 h of treatment with 40 nM of each IIQ revealed increased expression of γH2AX, suggestive of induced DNA damage, a primary anticipated consequence of TOP1 inhibition. In addition, treated cells demonstrated increased expression of cleaved Caspase 3, suggestive of induced extrinsic apoptosis. In the most sensitive cell lines, such as 5838 and RDES, we also observed induction of cleaved PARP1, a marker of late apoptosis (Figure 1B). To confirm DNA damage as a mechanism of action for IIQs, we performed comet assays in a subset of cell lines (ES4 and EW8), which were selected to represent both *TP53*- and *STAG2*-wild-type and mutated models. Using doses that resulted in diminished cell proliferation for each cell line (40 nM of each IIQ in ES4, 40 nM of LMP400 and LMP776 in ES4, and 80 nM of LMP744 in EW8), we observed increases in tail DNA percent following 24-h treatment with all IIQs in ES4 and with LMP400 and LMP776 in EW8 (Figure 1C). These results are consistent with the observed induction of γH2AX, indicating the ability of IIQs to induce DNA damage.

To characterize the resulting cell fate, we performed cell cycle analysis using the same concentrations of the IIQs described above. While we did not see significant treatment-induced differences in the proportion of cells in the G1, S, or G2/M phases for any of the IIQs, we did observe that LMP400 induced time-dependent accumulation of a sub-G1 phase population after 24-, 48-, and 72-h treatments in both ES4 and EW8. By 72 h post-treatment, we observed >10-fold increases in the sub-G1 phase population, relative to the control, with LMP400 and LMP776 in EW8, and with each IIQ in ES4, reflecting an increase in the proportion of non-viable cells (Figure 1D; Supplementary Figure S3). Assays of early and late apoptosis using Annexin V/PI staining similarly demonstrated that IIQ treatments induced time-dependent apoptosis in both cell lines. We observed an increase in the proportion of IIQ-treated cells with positive Annexin staining (early apoptosis) and double staining (late apoptosis) compared to control, confirming the mechanism of cell death (Figure 1E; Supplementary Figure S4). Collectively, these data suggest that IIQs effectively inhibit proliferation and induce DNA damage and apoptosis in EWS cells.

### Identification of IIQ maximum tolerated dose, optimal administration route, and dosing schedule *in vivo*

**FIGURE 2:**
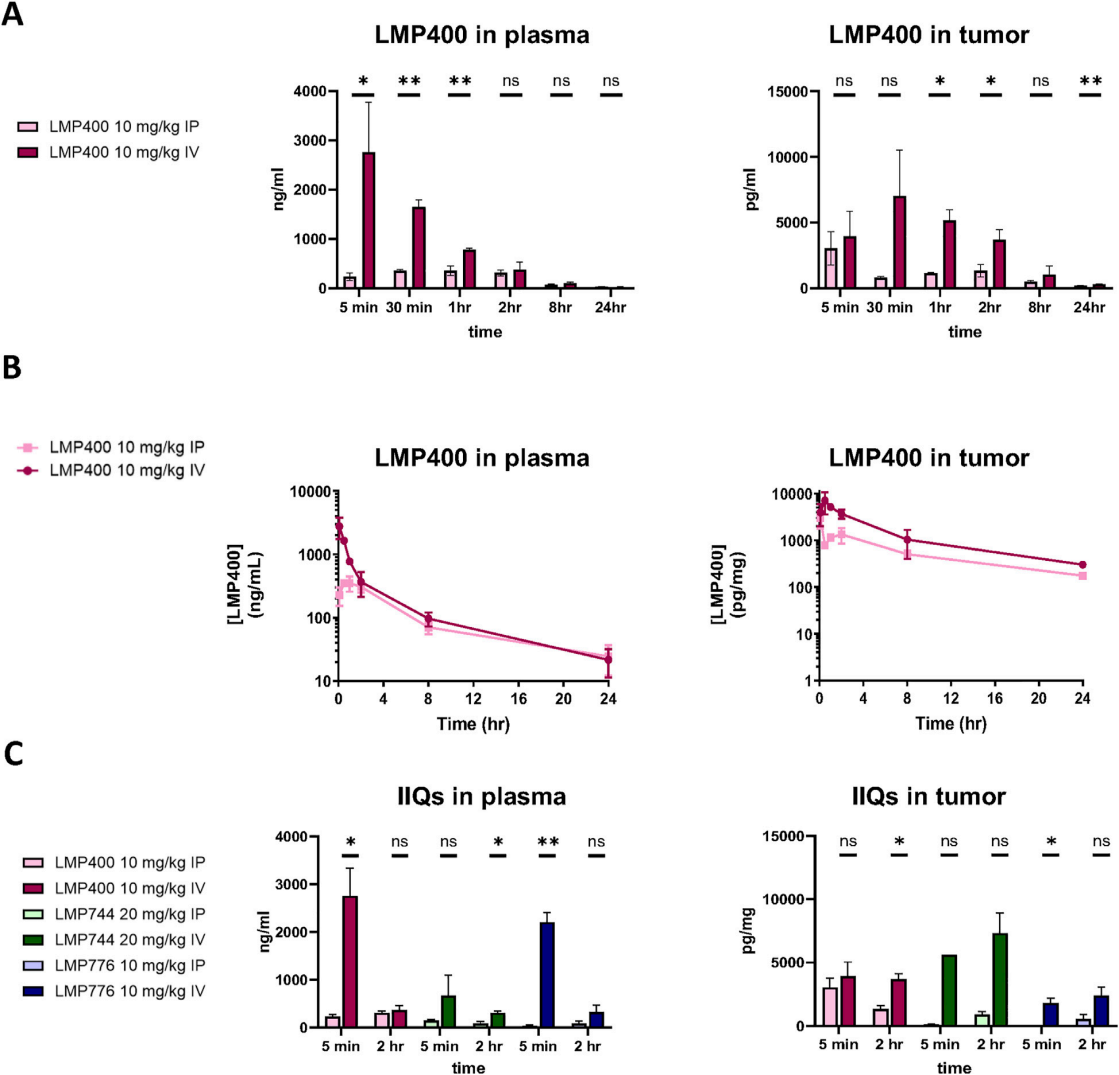
IV administration route results in higher plasma and tumor concentrations of the IIQs than IP route. **(A)**. Plasma (left panel) and tumor (right panel) levels of LMP400 following a single dose given either by IV or IP at 10 mg/kg at 5 min, 30 min, 1, 2, 8, and 24 h after treatment. **(B)**. Plasma (left panel) and tumor (right panel) levels of LMP400 administered IP or IV, plotted over time (5 min, 30 min, 1, 2, 8, and 24 h after treatment) for area under the curve (AUC) calculation. **(C)**. Plasma (left panel) and tumor (right panel) levels of LMP400 10 mg/kg, LMP744 20 mg/kg, LMP776 10 mg/kg following a single dose given either by IV or IP at 5 min and 2 h after treatment. Mann-Whitney test performed for each experiment (* indicates *p* ≤ 0.05, ** indicates *p* ≤ 0.01).

We next sought to test whether the *in vitro* efficacy observed with IIQs in EWS cell lines could be recapitulated *in vivo.* To determine the maximum tolerated dose (MTD) and optimal dosing regimen for each IIQ, we compared plasma and tumor pharmacokinetics of LMP400 delivered either by intravenous (IV) or intraperitoneal (IP) routes in mice bearing TC32 xenografts. We selected a dose of 10 mg/kg based on a previous report that this dose represented the MTD of LMP400 in an FVB mouse model (Marzi et al., 2019). Administration of LMP400 by the IV route resulted in significantly higher levels in plasma compared to IP administration within the first hour after dosing (*p* < 0.05 at 5 min; *p* < 0.01 at 30 min, and 1-hour post-dose). Similarly, in tumor tissue, IV administration achieved significantly higher levels of LMP400 at early time points (*p* < 0.05 at 1h, 2 h) as well as at the late time point (*p* < 0.01 at 24-hour-post dose) (Figure 2A). Accordingly, the area under the curve (AUC) for LMP400 was significantly higher with IV dosing compared to IP dosing both in plasma (*p* = 1.57 × 10^−8^) and in tumor (*p* = 1.912 × 10^−8^) (Figure 2B; Supplementary Table S5). The plasma and tumor half-life of LMP400 after IV and IP administration were similar (5.4 h versus 6 h in plasma; 7.5 versus 6.1 h in tumor).

To characterize the PK parameters for the other IIQs, a follow-up study comparing plasma and tumor drug levels after either IV or IP dosing for each IIQ at two time points (5 min and 2 h) was performed. Doses were again selected based on previously reported MTD data in FVB mice: 10 mg/kg for LMP400 and LMP776 and 20 mg/kg for LMP744 (Marzi et al., 2019). These data were concordant with the initial study, demonstrating that IV administration resulted in higher concentrations of each IIQ in plasma and in tumor compared to IP administration, with statistically significant differences observed in plasma at 5 min for LMP776 (*p* < 0.01) and LMP400 (*p* < 0.05), and at 2 h for LMP744 (*p* < 0.05), and in tumor at 5 min and 2 h in LMP776 (*p* < 0.05) and LMP400 (*p* < 0.05), respectively. IV administration of LMP400 and LMP776 resulted in the highest peak plasma concentrations, whereas the highest peak tumor concentrations were observed with LMP744 (Figure 2C), consistent with a recent study in dogs with naturally occurring lymphomas (Burton et al., 2018). Taken together, these results demonstrated that IV administration resulted in superior tumor exposure of IIQs compared to IP administration, establishing IV administration as the preferred route in this model.

Next, we conducted tolerability studies to determine the MTD values for the three IIQs by escalating doses previously established in an FVB model (Marzi et al., 2019). MTDs were determined in non-tumor bearing NSG mice by IV administration using a 5-day-on/2-day-off/5-day-on schedule (n = 5/condition). For all three IIQs we observed an MTD of 10 mg/kg, as increasing the dose resulted in systemic toxicity, excessive weight loss, or overt tail irritation (Supplementary Figure S5A). With the goal of increasing the dose level, we evaluated an altered treatment regimen with a longer dosing holiday (5-day-on/9-day-off/5-day-on). Tail irritation was noticeably improved on that regimen; however, 10 mg/kg was still the highest dose that did not result in weight loss or other systemic effects for all three compounds (Supplementary Figure S5B). Therefore, the 10 mg/kg 5-day-on/9-day-off/5-day-on dosing schedule was applied in subsequent efficacy studies.

### IIQs mediate antitumor effects in EWS PDX models

**FIGURE 3:**
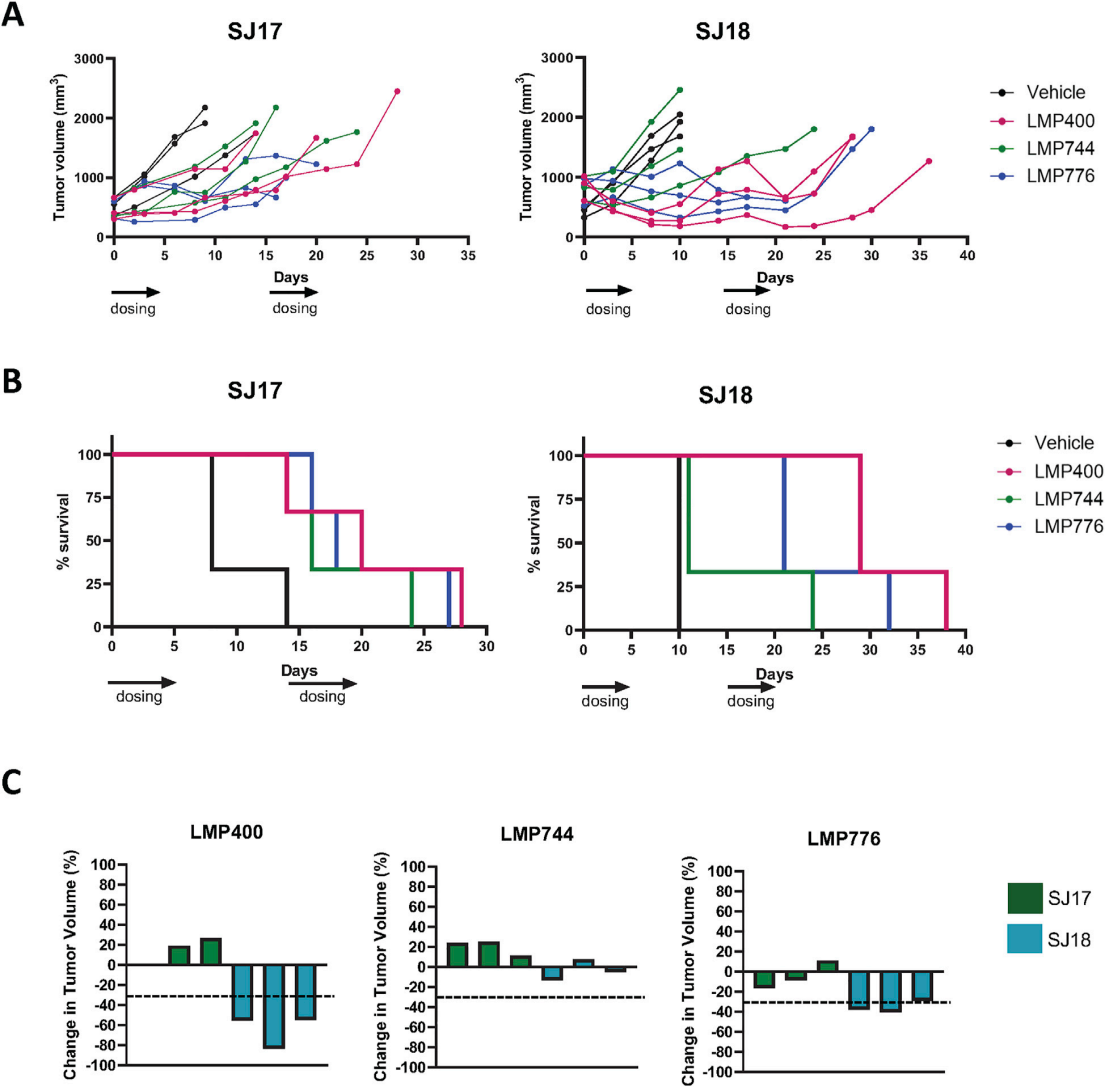
Pilot efficacy studies of IIQs in SJ17 and SJ18 PDX models show differential results. **(A)**. Spider plots for pilot efficacy studies (n = 3 mice/group) of vehicle (black), LMP400 (magenta), LMP744 (green), or LMP776 (blue) dosed IV at 10 mg/kg using a 5-day-on/9-day-off/5-day-on schedule in SJ18 and SJ17. Black arrows below *x*-axis indicate treatment days. **(B)**. Kaplan-Meier plots for pilot efficacy study of SJ17 and SJ18. Treatment groups were as listed for panel **(A)**. **(C)**. Waterfall plots showing best response in tumor volume (percent change) for each mouse enrolled in the pilot efficacy studies in SJ17 and SJ18 PDX models. Dashed line indicates the threshold for tumor regression of ≥30% that defines a response per RECIST.

Using the MTD established in the prior studies, we next conducted small pilot studies testing the efficacy of LMP400, LMP744, and LMP776 in two different PDX models, SJEWS-17-06841 (SJ17) and SJEWS-18-09520 (SJ18), using n = 3 mice/condition. Results from these pilot studies demonstrated that in both models, each IIQ slowed tumor growth rate or regressed tumors and consequently prolonged survival relative to the vehicle treatment group (Figures 3A, B). Interestingly, we observed a differential degree of tumor response to each IIQ between models. Specifically, in SJ17, LMP776 resulted in slight, non-sustained tumor regression in 2/3 mice, whereas LMP400 and LMP744 did not induce any tumor regression. In contrast, in SJ18, both LMP400 and LMP776 induced tumor regression that was sustained throughout the treatment period in most mice, despite the discontinuation of treatment of two mice in the LMP776 group due to weight loss; LMP744 had a more modest growth inhibitory effect in this model.

As tumor growth rate inhibition in animal models rarely translates to meaningful drug activity in clinical trials (Kim and Sharpless, 2012), we next sought to define a more stringent response criteria. Using the Response Evaluation Criteria in Solid Tumors (RECIST) of tumor regression of ≥30% to define a response (Schwartz et al., 2016), we calculated an average best response for each PDX model, as responses to each agent were generally concordant for all mice bearing the same PDX (Figure 3C). Based on these criteria, SJ17 was characterized as a non-responder to all three IIQs, whereas SJ18 was characterized as a responder to both LMP400 and LMP776, but not to LMP744. Among the IIQs, LMP400 was the most tolerated and efficacious in these pilot studies, and thus, we prioritized this agent for subsequent studies.

### EWS PDX models demonstrate differential responses to LMP400 and irinotecan

Since our pilot studies included a small number of mice per group (n = 3/condition), we next repeated the LMP400 experiments with larger cohorts of mice (n = at least 10/condition) to confirm the pilot findings. In addition, given the differential responses to IIQs in our two pilot models, we expanded the efficacy studies to include the two pilot models plus four additional molecularly and clinically heterogeneous EWS PDX models: SJEWS-17-06841 (SJ17), SJEWS-18-09520 (SJ18), XEN-EWS-021 (NCI21), NCH-EWS-4 (NCH4), NCH-EWS-1 (NCH1), and SJEWS049193_X1 (SJ49). In addition to a vehicle control, we included an irinotecan arm for comparison to standard TOP1 inhibitor therapy. Irinotecan treatments were administered IP at a clinically relevant dose of 2.5 mg/kg, while LMP400 was delivered using the same dose as in the pilot experiments. All treatments were administered on a 5-day-on/9-day-off/5-day-on schedule.

**FIGURE 4:**
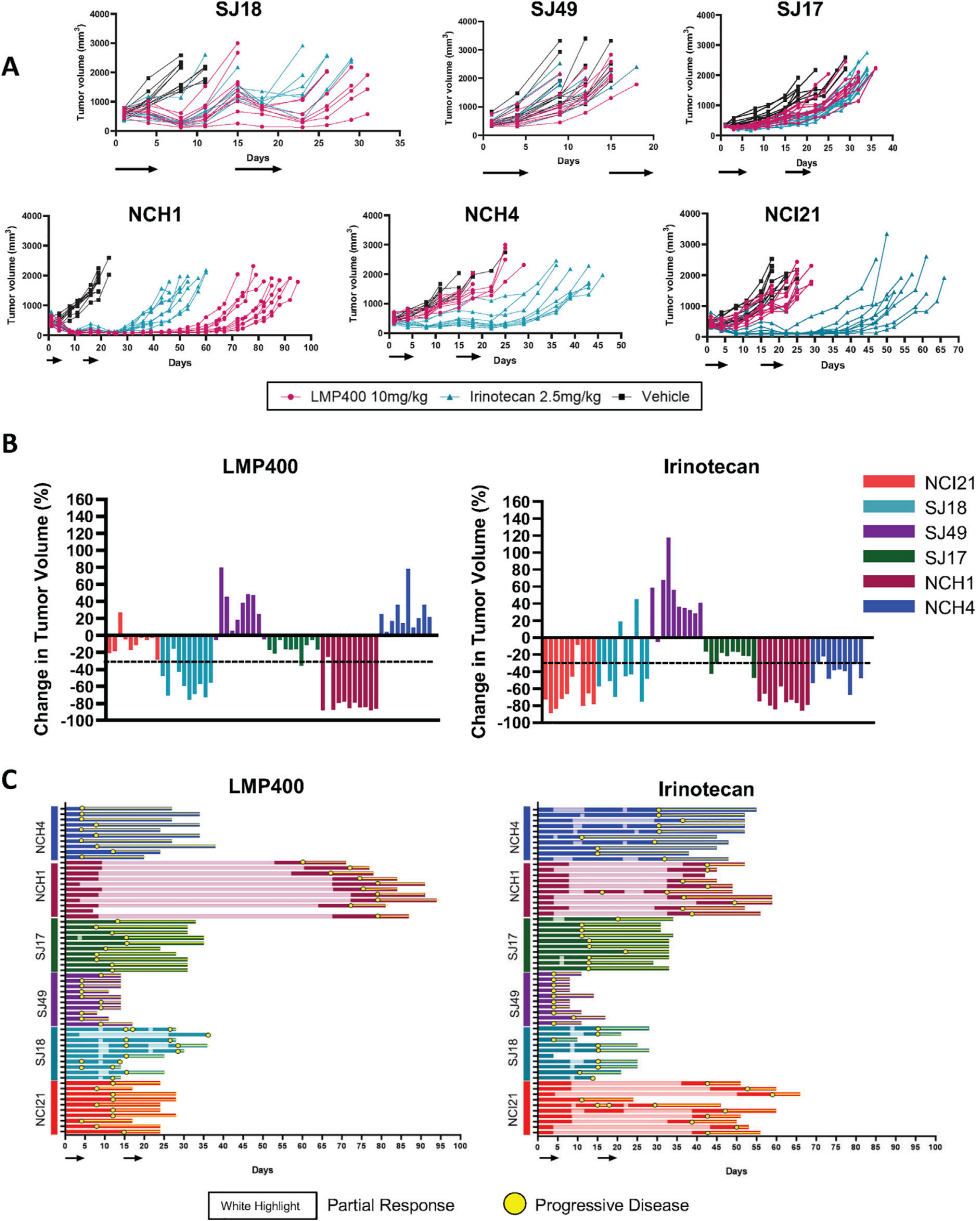
Expanded efficacy studies of LMP400 in six EWS PDX models show differential results. **(A)**. Spider plots for expanded efficacy studies (n = 10 mice/group) of vehicle (black), LMP400 (magenta) dosed IV, and irinotecan dosed IP at 2.5 mg/kg (teal) using a 5-day-on/9-day-off/5-day-on schedule in SJ18, SJ49, SJ17, NCH1, NCH4, NCI21. Black arrows below *x*-axis indicate treatment days. **(B).** Waterfall plot showing best response in tumor volume (percent change) for each mouse enrolled in the expanded efficacy studies for LMP400 (left panel) and irinotecan (right panel) in six EWS PDX models. Dashed line indicates the threshold for tumor regression of ≥30% that defines a response per RECIST. **(C)**. Swimmer plot showing survival of each mouse enrolled in the expanded efficacy studies of LMP400 (left panel) and irinotecan (right panel) in six EWS PDX models. Yellow circles indicate time points at which at least 20% tumor growth was observed (progression) while the white highlight indicate time points at which at least 30% tumor reduction was observed (response).

Results for the two models used in the pilot study were concordant with those from this larger follow-up experiment, with 9 of 10 mice bearing the SJ18 PDX exhibiting a partial response to LMP400 and 9 of 10 mice bearing the SJ17PDX not responding. Across the larger panel of PDX models, we observed a wide range of responses to both LMP400 and irinotecan (Figure 4A). In all but one model (SJ49), both drugs prolonged survival, albeit to differing degrees (Supplementary Figure S6). In line with clinical response criteria (Schwartz et al., 2016), we again defined a responder as a model with an average best response of tumor shrinkage ≥30%. Based on these criteria, we determined that two of six models were non-responders to both drugs (SJ17 and SJ49); two of six models were responders to both drugs (NCH1 and SJ18), and two of six models were non-responders to LMP400 but responders to irinotecan (NCI21 and NCH4) (Figure 4B; Table 1). In addition, we analyzed the duration of response for each model (Figure 4C; Supplementary Figure S6). In both models that responded to both LMP400 and irinotecan, LMP400 induced a more durable effect. In the NCH1 model, the longest sustained tumor response in the LMP400 group was 52 days after the end of treatment, whereas in the irinotecan-treated group, it was just 19 days. Survival analysis of the irinotecan- and LMP400-treated animals also demonstrated a statistically significant difference in survival between the groups (*p* < 0.0001) with median survival of 49 versus 84 days. In the SJ18 model, the durability effect was more modest. Irinotecan-treated mice experienced progressive disease (≥20% tumor growth) by the second half of the treatment cycle, whereas a subset (4 of 10) LMP400-treated mice still demonstrated tumor regression (≥30% tumor reduction) during that period. Survival analysis demonstrated that LMP400-treated mice experienced longer median survival (27 days) compared to irinotecan-treated mice (21 days) although this difference did not reach statistical significance (*p* = 0.512) and may not reflect a clinically meaningful difference.

**TABLE 1:** Summary of PDX responses to LMP400 and irinotecan.

PDX model	Abbreviation	LMP400 average best response	LMP400 response^a^	Irinotecan average best response	Irinotecan response^a^
XEN-EWS-021	NCI21	−8.37%	Non-responder	−65.96%	Responder
NCH-EWS-1	NCH1	−78.48%	Responder	−75.16%	Responder
NCH-EWS-4	NCH4	n/a	Non-responder	−41.30%	Responder
SJEWS-17-06841	SJ17	−15.97%	Non-responder	−25.06%	Non-responder
SJEWS-18-09520	SJ18	−56.44%	Responder	−35.40%	Responder
SJEWS-049193_X1	SJ49	n/a	Non-responder	n/a	Non-responder

^a^ Responder indicates ≥30% tumor reduction; n/a indicates no tumor shrinkage.

### Transcriptomic analysis reveals biomarker gene signature of LMP400 responsiveness in EWS PDX models

In spite of the small sample size, to interrogate a potential molecular basis for the mixed set of drug responses, we performed genomic analyses including whole genome and bulk RNA sequencing of each PDX model. The full dataset has been uploaded to a publicly available web tool, the Ewing Sarcoma MinerCDB (https://discover.nci.nih.gov/rsconnect/EwingSarcomaMinerCDB/) (Tlemsani et al., 2024). Table 2 summarizes the characteristic EWS fusions as well as the molecular features of each PDX that have been commonly described as prognostic in EWS patient tumors (Shulman et al., 2022), including *TP53* and *STAG2* mutational status (Supplementary Dataset S2). We did not observe a correlation between tumor response to LMP400 and either of these mutations, nor did we identify any other mutations that correlated with response.

**TABLE 2:** Molecular characteristics of PDX models.

PDX model	Abbreviation	Fusion	TP53	STAG2
XEN-EWS-021	NCI21	*EWSR1::FLI1*	Homozygous mutation	Mutation present^a^
NCH-EWS-1	NCH1	*EWSR1::FLI1*	WT	WT
NCH-EWS-4	NCH4	*EWSR1::FLI1*	WT	WT
SJEWS-17-06841	SJ17	*EWSR1::FLI1*	Homozygous mutation	Homozygous mutation
SJEWS-18-09520	SJ18	*EWSR1::FLI1*	WT	Heterozygous mutation
SJEWS-049193_X1	SJ49	*EWSR1::FLI1*	WT	WT

^a^ Mutation present indicates evidence of mutation with variant allele frequency less than 50%.

Turning to gene expression, we first examined the expression of Schlafen 11 (*SLFN11*), which is known to be a dominant determinant of response to TOP1 inhibitors (Marzi et al., 2019; Zoppoli et al., 2012; Barretina et al., 2012) and is highly expressed in EWS (Garnett et al., 2012) due to its transcriptional activation by FLI1 (Tang et al., 2015). As expected, *SLFN11* was highly expressed in all PDX samples with a relatively narrow range of expression and reproducible values across each of the triplicate determination. Although the levels of *SLFN11* expression shared some overlap between responder and non-responder models, we nevertheless observed a positive correlation between the activity of LMP400 and *SLFN11* expression across these models (Supplementary Figure S7A).

**FIGURE 5:**
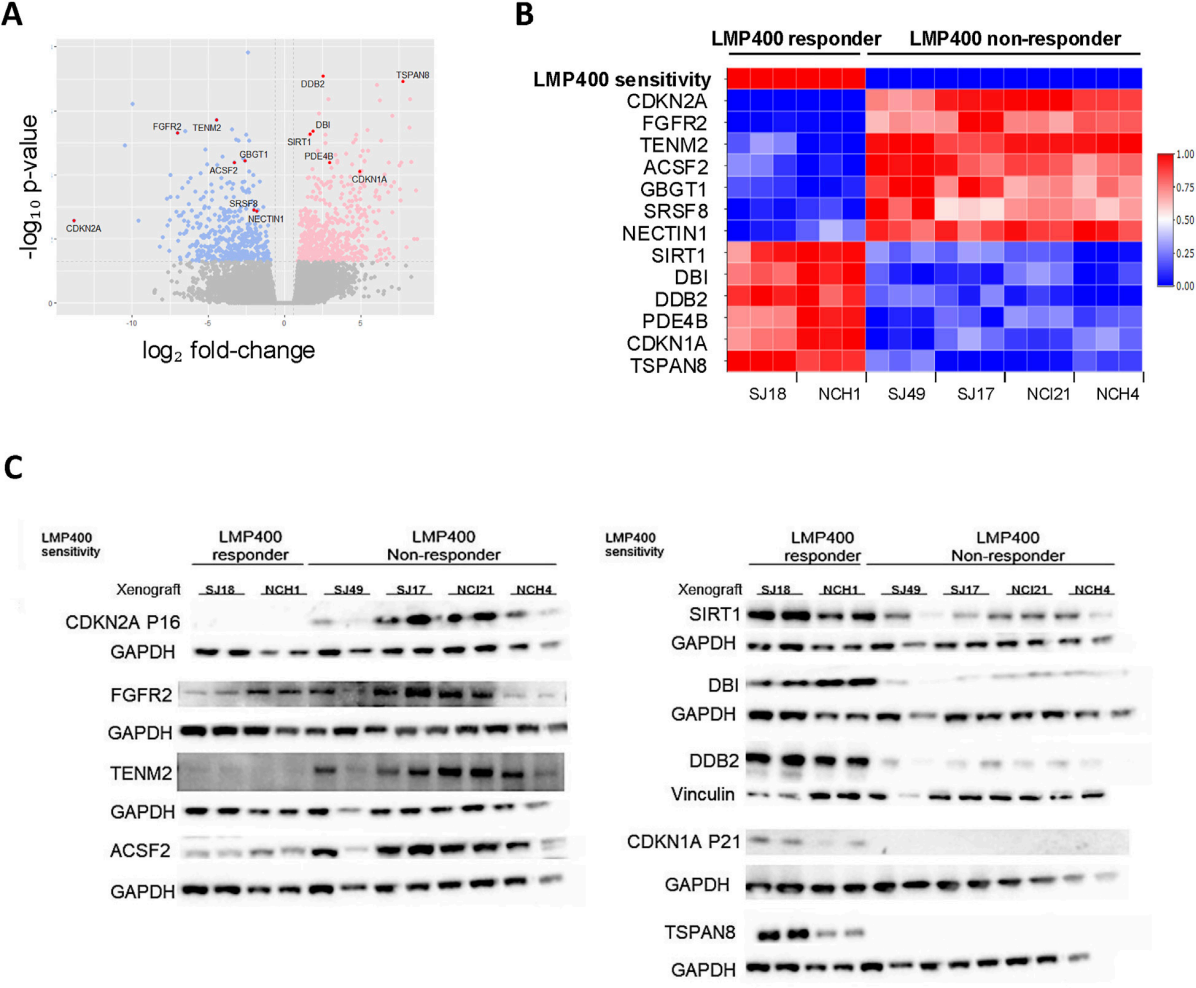
Transcriptomic analysis comparing LMP400-responder and non-responder EWS PDX models reveals a potential predictive gene signature of LMP400 response. **(A)**. Volcano plot showing differentially expressed genes between LMP400-responder versus LMP400-non-responder EWS PDX models as analyzed with limma + voom in limma package. Blue coloring indicates downregulated genes in responder PDXs relative to non-responder PDXs; pink coloring indicates upregulated genes using the same comparison. Significance was defined as |log_2_FC| > 0.6 with *p*-value <0.05. Genes colored in grey are not significantly differentially expressed. The 13 biomarkers of interest identified using the LASSO feature selection algorithm are highlighted in red and labeled. **(B)**. Heatmap showing normalized RNA expression levels for the top 13 candidate biomarker genes identified from our screening process. Figure reflects data from three independent biological replicates for each PDX. **(C)**. Western blot analysis showing the protein expression of our top 13 biomarkers of interest. At least two biological replicates for each PDX were included. *TENM2*, *SRSF8* (shown in Supplementary Figure S7), and *NECTIN1* (shown in Supplementary Figure S7) were probed on the same membrane; *ACSF2*, *GBGT1* (shown in Supplementary Figure S7), and *TSPAN8* were probed on the same membrane. Antibodies probed on the same membrane display the same loading control.

Using the LASSO feature selection algorithm (Luna et al., 2021) and differential gene expression analysis (Limma), we identified the most differentially expressed genes (DEGs) between responder and non-responder models. This revealed a candidate biomarker signature of 13 genes: *CDKN2A, FGFR2, TENM2, ACSF2, GBGT1, SRSF8, NECTIN1, SIRT1, DBI, DDB2, PDE4B, CDKN1A,* and *TSPAN8.* (Figures 5A, B). Lack of *CDKN2A* expression alone was also found to be highly predictive for LMP400 response (Supplementary Figure S7B) and to be epigenetically driven *via* promoter methylation (Supplementary Figure S7C). Evaluation of protein expression for each candidate gene demonstrated concordance between the RNA expression and protein expression for 9 of the 13 genes. For the other four genes, *GBGT1, SRSF8, NECTIN1*, and *PDE4B*, RNA expression and protein expression were not correlated, suggesting there may be post-transcriptional regulation of these genes (Figure 5C; Supplementary Figure S8). While this collective set of 13 genes may represent a potential biomarker signature predicting LMP400 sensitivity for EWS, further validation using samples from EWS patients treated with LMP400, preferably in the context of a clinical trial, is necessary. In addition, the specific mechanistic role of each of these genes on LMP400 sensitivity in EWS remains an area for future research.

## Discussion

In this report, we characterized the PK, toxicity, and response pattern to the novel TOP1 inhibitors, IIQs, in preclinical models of EWS. Our study demonstrated that *in vitro*, IIQs exhibited anti-proliferative effects at nanomolar concentrations across each of the eight EWS cell lines tested, inducing DNA damage and apoptotic cell death, independent of *STAG2* and *TP53* mutational status. *In vivo,* we noted heterogeneous responses to LMP400 (indotecan) across a panel of six molecularly diverse PDX models. We found two of six PDXs undergoing tumor regression ≥30% and four of six PDXs with a best response of decreased tumor growth rate, but no regression. Comparisons to the standard of care TOP1 inhibitor irinotecan revealed that LMP400 resulted in tumor regression in fewer PDX models, suggesting a potential therapeutic liability of this class of agents in this disease. However, the duration of regression was substantially longer with LMP400 than irinotecan in one of the models, demonstrating that tumor responsiveness to these agents is not straightforward. In addition, we identified a candidate biomarker signature based on Lasso and differential gene expression between PDX models that underwent regression versus those that did not. The genomic data from the PDX models are available at the Ewing Sarcoma MinerCDB (https://discover.nci.nih.gov/rsconnect/EwingSarcomaMinerCDB/).

The predictive value of preclinical drug testing for anticancer agents remains complex, as many agents with seemingly promising preclinical activity ultimately fail when translated into clinical trials (Kim and Sharpless, 2012). Our results highlight some of the challenges of preclinical drug testing, including the fact that agents that may be broadly efficacious in numerous cancer cell lines demonstrate relatively limited activity in *in vivo* models. Conventional cell lines lack the ability to demonstrate high predictive value for future clinical activity, and yet they form the basis of most studies evaluating anticancer agents (Hidalgo et al., 2014; Gillet et al., 2011). *In vivo* models, while considered potentially more predictive than *in vitro* models, are frequently derived from these same cell lines. To mitigate this limitation, we utilized a panel of PDX models for our *in vivo* studies, which better recapitulate biological properties specific to the cancer type as well as greater tumor heterogeneity, and therefore, may be more representative disease models (Tentler et al., 2012; Siolas and Hannon, 2013). Indeed, in our study, we observed a surprising range of heterogeneity in response to both TOP1-targeting chemotherapeutic agents, LMP400 and the standard of care agent irinotecan, across the six PDX models tested. These results underscore the importance and the feasibility of including a broad range of molecularly distinct models within the same disease type in *in vivo* studies. Encouragingly, we found that the results from our pilot experiments with a few mice per group were concordant with the results from our larger experiments. This observation suggests that prioritizing resources to test a greater variety of PDX models with fewer animals per model is a viable strategy going forward.

A second limitation to the predictive value of preclinical results to clinical translation may be the benchmarks typically used for defining activity in animal studies. Many studies define an agent as active if it reduces tumor growth rate or prolongs animal survival compared to an untreated control (Gengenbacher et al., 2017; Talmadge et al., 2007). However, in clinical practice, a mere slowing of tumor progression would not constitute an objective response (Schwartz et al., 2016; Gengenbacher et al., 2017). Accordingly, we applied a more stringent response criteria for our *in vivo* models than is typically used in such studies, using the same response metric that is used for early phase clinical studies, >30% tumor shrinkage, to define a response. While five of the six models showed statistically significant improvements in survival with LMP400, just two of six models showed a response using these stricter criteria. Furthermore, by including an irinotecan arm as a standard of care benchmark comparison in these studies, we have established study conditions that may provide additional translational relevance. Data are scarce regarding the performance of standard of care drugs in PDX models, since such models were not accessible at the time that current standard treatments were tested (Pusch et al., 2024). In our studies, we observed that while five of six models showed statistically significant improvements in survival with clinically relevant doses of irinotecan, only four of six models showed a response using the regression criteria. Indeed, clinical reports of the efficacy of irinotecan in patients with EWS demonstrate response rates of approximately 40%–60%, which is in line with what we observed in our models (Raciborska et al., 2013; Casey et al., 2009; Bisogno et al., 2006; Cosetti et al., 2002; Vassal et al., 2007; Wagner et al., 2004; Wagner et al., 2005; McNall-Knapp et al., 2010; Wagner et al., 2010; Wagner et al., 2013; Venkatramani et al., 2013). Finally, we assessed the durability of response as an independent metric from survival and found that even in responding models, there was wide variability in length of response. In one of the two models that responded to LMP400 and irinotecan, the response duration was significantly longer with LMP400, suggesting that for some EWS tumors, there is a potential advantage of this agent over the standard TOP1 inhibitor.

As modern cancer medicine has undergone a paradigm shift from a ‘one drug fits all’ concept to a more personalized strategy, there has been an emphasis on identifying biomarkers of response early in the clinical development process. Predictive biomarkers of response can be used to guide patient selection for clinical trials, thus maximizing the potential for clinical benefit to study participants (La Thangue and Kerr, 2011). This has proven challenging in rare diseases like pediatric-type sarcomas, where, in contrast to numerous adult malignancies, therapy is rarely biomarker driven (Shukla et al., 2013; Goldman et al., 2011). Studies examining predictive biomarkers for camptothecin derivatives in various cancer cell lines have identified high *SLFN11* expression as a biomarker of response to DNA damaging agents, including IIQs (Marzi et al., 2019; Zoppoli et al., 2012; Barretina et al., 2012; Lok et al., 2017). In EWS however, *SLFN11* is known to be highly expressed (Tang et al., 2015; Gartrell et al., 2021) with little variability, which may limit its utility as a biomarker. Similarly, *STAG2* loss and *TP53* mutations are other molecular biomarkers that have been shown to influence therapeutic responsiveness in various cancer types, including EWS (de Alava et al., 2000; Robles and Harris, 2010; Brohl et al., 2014). Considering the complexity of the role of these mutations in different cancer types and in the context of specific biology, further studies may elucidate the potential relevance of these prognostic biomarkers to LMP400 response in EWS.

Using molecular and response data from our PDX panel and the Ewing Sarcoma MinerCDB analysis package released with this publication (https://discover.nci.nih.gov/rsconnect/EwingSarcomaMinerCDB/), we have identified a preclinical candidate gene signature for predicting response to LMP400 in EWS. Acknowledging that a single genetic feature alone may not be sufficient to explain complex phenotypes, including drug response, that a lack of downstream functional analyses limits definitive conclusions regarding a singular causative mechanistic explanation contributing to drug response, and that preclinical models have limitations, we propose, as a next step, trying to validate this biomarker signature in the context of an early phase clinical trial testing LMP400 in patients with EWS. If successful, this approach of using a PDX library to derive biomarker signatures of response to novel agents in rare cancers could serve as a model for biomarker discovery for novel agents in the future.

In summary, our results demonstrate that IIQs, a novel class of topoisomerase inhibitors with a clinically improved toxicity profile, may be promising agents for a subset of EWS patients. Additional preclinical work and correlative biology studies in conjunction with early phase clinical investigation will be required to fully assess their translational potential.

## Data Availability

The data included in this study are available within the supplemental data files, and at the Ewing Sarcoma MinerCDB site: https://discover.nci.nih.gov/rsconnect/EwingSarcomaMinerCDB/. Additional data are available upon request from the corresponding author.
