# Supplementary material for: Treatment with novel topoisomerase inhibitors in Ewing sarcoma models reveals heterogeneity of tumor response
Source: Front Cell Dev Biol. 2024 Oct 24;12:1462840. doi: 10.3389/fcell.2024.1462840 (PMC11542432; doi:10.3389/fcell.2024.1462840)
Supplement: Supplementary file 1 [file Table5.pdf]

Treatment with novel topoisomerase inhibitors in Ewing sarcoma models reveals heterogeneity of tumor response

Unsun Lee, Ludmila Szabova, Victor J. Collins, Melanie Gordon, Kristine Johnson, Deborah Householder, Stephanie Jorgensen, Lucy Lu, Laura Bassel, Fathi Elloumi, Cody J. Peer, Ariana E. Nelson, Sophia Varriano, Sudhir Varma, Ryan D. Roberts, Zoe Weaver Ohler, William D. Figg, Shyam K. Sharan, Yves Pommier, Christine M. Heske

Supplemental Table S5. PK Parameter Data for Plasma (A) and Tumor (B)

A.

		LMP400 (10 mg/kg IP)						LMP400 (10 mg/kg IV)					
PLASMA	**Time (hr)**	**0.083**	**0.5**	**1**	**2**	**8**	**24**	**0.083**	**0.5**	**1**	**2**	**8**	**24**
	**Plasma Conc (ng/mL)**	256.2	329.3	377.4	310.9	60.3	12.9	3585.3	1634.6	753	397.8	71.2	31.4
		285.4	338.3	248.2	366.9	62.6	23.1	1635.5	1797	813.2	199	100.5	11.1
		144.2	379.3	430.1	249	88.5	37.4	3058.3	1510.2	755.8	510.4	118.8	22.1
	**Mean**	180.53	222.71	208.86	226.59	43.63	19.99	1740.3	1144	522.4	199.61	59.92	22.13
	**T1/2 (hr)**	6.011						5.363					
	**AUC ((hr*ng)/mL)**	2523.6						4440.4					
	**Clearance (mL/hr)**	79.25						45.04					

B.

		LMP400 (10 mg/kg IP)						LMP400 (10 mg/kg IV)					
TUMOR	**Time (hr)**	**0.083**	**0.5**	**1**	**2**	**8**	**24**	**0.083**	**0.5**	**1**	**2**	**8**	**24**
	**Tumor Conc (pg/mL)**	4357.2	736.2	1202.2	1121	379.6	172	6071.7	10181.8	5429.3	4466.7	1718.5	290.8
		1845.3	904.1	1112.6	1867.4	565.2	155.5	3399.2	3307.1	4226.7	2864.1	478.1	284.7
		2886.4	703.4	1089.7	996.5	564.1	199.4	2339.2	7609	5769	3668.3	899.8	332.2
	**Mean**	3029.64	781.24	1134.83	1328.31	502.97	175.62	3936.69	7032.62	5141.65	1032.37	1032.09	302.54
	**T1/2 (hr)**	7.535						6.111					
	**AUC ((pg*hr)/mg)**	13427.7						34507.1					
