# Supplementary material for: Treatment with novel topoisomerase inhibitors in Ewing sarcoma models reveals heterogeneity of tumor response
Source: Front Cell Dev Biol. 2024 Oct 24;12:1462840. doi: 10.3389/fcell.2024.1462840 (PMC11542432; doi:10.3389/fcell.2024.1462840)
Supplement: Supplementary file 2 [file Table4.pdf]

Treatment with novel topoisomerase inhibitors in Ewing sarcoma models reveals heterogeneity of tumor response

Unsun Lee, Ludmila Szabova, Victor J. Collins, Melanie Gordon, Kristine Johnson, Deborah Householder, Stephanie Jorgensen, Lucy Lu, Laura Bassel, Fathi Elloumi, Cody J. Peer, Ariana E. Nelson, Sophia Varriano, Sudhir Varma, Ryan D. Roberts, Zoe Weaver Ohler, William D. Figg, Shyam K. Sharan, Yves Pommier, Christine M. Heske

Supplemental Table S4. Molecular characteristics of EWS cell lines. (1, 2)

Cell Line	Fusion Type	TP53	STAG2
**EW8**	EWS-FLI1 Type I	Y220C- No protein	N475fs- No protein
**TC71**	EWS-FLI1 Type I	R213X & G245C- No protein	
**TC32**	EWS-FLI1 Type I		Y636fs- No protein
**RDES**	EWS-FLI1 Type II	R273C	
**5838**	EWS-ERG	Unknown	Unknown
**ES1**	EWS-FLI1 Type II	R248Q	
**ES4**	EWS-FLI1 Type II		
**ES6**	EWS-FLI1 (9/4)	Expression loss	L264P

1. Brohl, A.S., et al., *The genomic landscape of the Ewing Sarcoma family of tumors reveals recurrent STAG2 mutation.* PLoS Genet, 2014. **10**(7): p. e1004475.
2. Dunn, T., L. Praissman, N. Hagag, and M.V. Viola, *ERG gene is translocated in an Ewing's sarcoma cell line.* Cancer Genet Cytogenet, 1994. **76**(1): p. 19-22.
