# Supplementary material for: Treatment with novel topoisomerase inhibitors in Ewing sarcoma models reveals heterogeneity of tumor response
Source: Front Cell Dev Biol. 2024 Oct 24;12:1462840. doi: 10.3389/fcell.2024.1462840 (PMC11542432; doi:10.3389/fcell.2024.1462840)
Supplement: Supplementary file 3 [file Image5.pdf]

Supplemental Figure S5

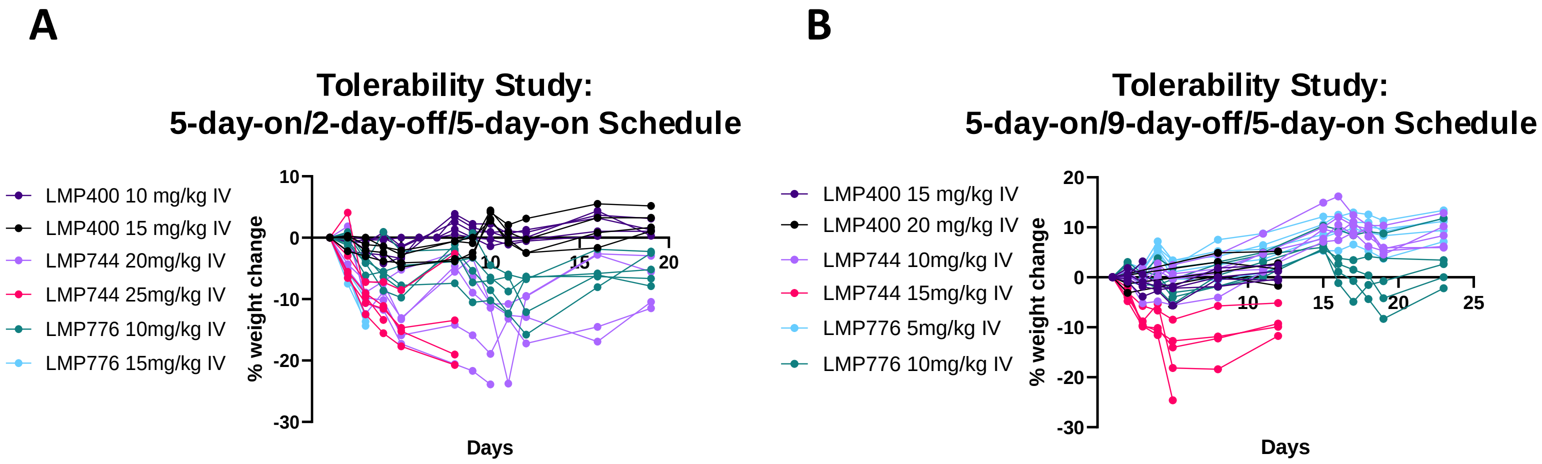

Supplemental Figure S5. Effect of IIQs on animal weights. A. Spider plot showing percent weight change in each mouse in a tolerability study testing IIQs administered by IV tail injection in a 5-day-on/2-day-off/5-day-on dosing schedule. Besides dramatic weight loss observed with higher doses of LMP744 and systemic toxicity with higher dose of LMP776, tail irritation was observed in majority of groups. **B**. Spider plot showing percent weight change in each mouse in a tolerability study testing IIQs administered by IV tail injection in a 5-day-on/9-day-off/5-day-on dosing schedule. Higher doses (>10mg/kg) of LMP400 and LMP744 resulted in systemic toxicity.
