# Supplementary material for: Treatment with novel topoisomerase inhibitors in Ewing sarcoma models reveals heterogeneity of tumor response
Source: Front Cell Dev Biol. 2024 Oct 24;12:1462840. doi: 10.3389/fcell.2024.1462840 (PMC11542432; doi:10.3389/fcell.2024.1462840)
Supplement: Supplementary file 4 [file Table1.pdf]

Treatment with novel topoisomerase inhibitors in Ewing sarcoma models reveals heterogeneity of tumor response

Unsun Lee, Ludmila Szabova, Victor J. Collins, Melanie Gordon, Kristine Johnson, Deborah Householder, Stephanie Jorgensen, Lucy Lu, Laura Bassel, Fathi Elloumi, Cody J. Peer, Ariana E. Nelson, Sophia Varriano, Sudhir Varma, Ryan D. Roberts, Zoe Weaver Ohler, William D. Figg, Shyam K. Sharan, Yves Pommier, Christine M. Heske

Supplemental Table S1. Key Reagents

Reagent	Identifier and Other Information	Source
**Compounds/Drugs**		
LMP400	TOP1 inhibitor	DTP, NCI
LMP744	TOP1 inhibitor	DTP, NCI
LMP776	TOP1 inhibitor	DTP, NCI
Irinotecan	TOP1 inhibitor	NIH Veterinary Pharmacy
**Antibodies**		
GAPDH-HRP	Sc-47724 (1:2000)	Cell Signaling Technology (CST)
Vinculin	13901 (1:2000)	CST
PARP1	#9542 (1:1000)	CST
Caspase 3	#9662 (1:1000)	CST
Γ-H2AX	#80312 (1:1000)	CST
P16 INK4A	#80772 (1:1000)	CST
SIRT1	#9475 (1:1000)	CST
DDB2	#5416 (1:1000)	CST
PDE4B	#72096 (1:500)<br>24h extended incubation	CST
P21	#2947 (1:1000)	CST
FGFR2	#6930 (1:500)<br>24h extended incubation	Santa Cruz
NECTIN1	37-5900 (1:1000)	Thermo Fisher Scientific
DBI	144490-1-AP (1:1000)	Proteintech
TSPAN8	Ab70007 (1:1000)	Abcam
TENM2	AF4578-SP (1:1000)	R&D Systems
ACSF2	16140-1-AP (1:1000)	Proteintech
GBGT1	PA5-116392 (1:1000)	Thermo Fisher Scientific
SRSF8	PA5-41917 (1:1000)	Thermo Fisher Scientific
Anti-mouse	7076S	CST
Anti-rabbit	7074S	CST
Anti-sheep	#A16041	Thermo Fisher Scientific
CD99	MA5-16416 (1:500)	Thermo Fisher Scientific
**Chemicals (in-vitro)**		
RPMI Medium	21870-084	Thermo Fisher Scientific
Fetal Bovine Serum	12306C-500ML	Sigma Aldrich
Penicillin-Streptomycin	15140-122	Thermo Fisher Scientific
L-glutamine (100X)	25030-081	Thermo Fisher Scientific
Trypsin-EDTA (0.25%)	25200-056	Thermo Fisher Scientific
Trypsin-EDTA (0.05%)	25300-054	Thermo Fisher Scientific
Protease Inhibitor Cocktail	78446	Thermo Fisher Scientific

1X RIPA Lysis and Extraction Buffer	89901	Thermo Fisher Scientific
4X LDS Sample Loading Buffer	NP0007	Thermo Fisher Scientific
10X Sample Reducing Agent	NP0009	Thermo Fisher Scientific
Skim Milk Powder	Sc-2325	ChemCruz
SuperSignal West Pico Chemiluminescent substrate	34580	Thermo Fisher Scientific
SuperSignal West Femto Chemiluminescent substrate	34096	Thermo Fisher Scientific
Tween20	P1379-500ML	Sigma Aldrich
MOPS SDS Running Buffer (20X)	NP0001	Thermo Fisher Scientific
NuPAGE Transfer Buffer (20X)	NP00061	Thermo Fisher Scientific
Tris Buffered Saline, 10 X	5460-0037	SeraCare Life Sciences
0.1 % Triton X-100		Millipore Sigma
Propidium Iodide	P4864-10ML	Sigma Aldrich
Phosphate buffered Saline	14190-144	Thermo Fisher Scientific
DNAse-free RNAse A	R5503	Millipore Sigma
T-PER Tissue Protein Extraction Reagent	#78510	Thermo Fisher Scientific
**Chemicals (in-vivo)**		
Hank's balanced salt solution	14025134	ThermoFisher Scientific
HCl	H1758	Sigma Aldrich
Dextrose	D9434	Sigma Aldrich
Matrigel	354248	Corning
Citric acid	1.93026	Sigma Aldrich
**Commercial Assays**		
BCA Kit	23225	Thermo Fisher Scientific
AllPrep DNA/RNA Micro Kit	#80284	Qiagen
RNase-Free DNase Set	#79254	Qiagen
TissueRuptor II system		Qiagen
CometAssay Single Cell Assay	4250-050-K	Bio-Techne
Annexin V-FITC Assay Kit	600300	Cayman Chemical Company
