# Supplementary material for: Treatment with novel topoisomerase inhibitors in Ewing sarcoma models reveals heterogeneity of tumor response
Source: Front Cell Dev Biol. 2024 Oct 24;12:1462840. doi: 10.3389/fcell.2024.1462840 (PMC11542432; doi:10.3389/fcell.2024.1462840)
Supplement: Supplementary file 6 [file Image6.pdf]

Supplemental Figure S6

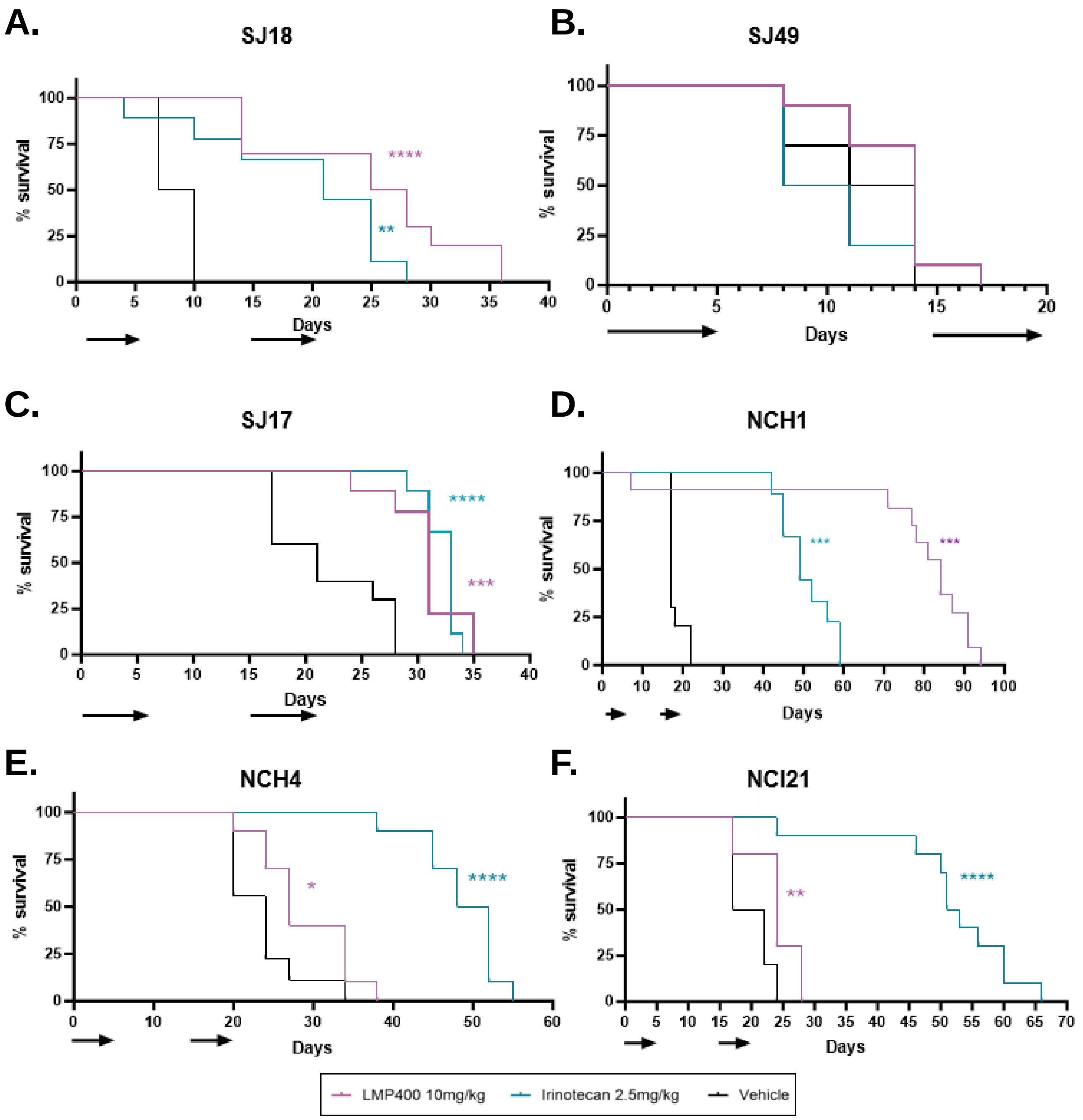

Supplemental Figure S6. LMP400 10 mg/kg IV treatment administered on a 5-day-on/9-day-off/5-day-on treatment cycle prolongs survival compared to vehicle in 5 of 6 EWS PDX models. Kaplan-Meier survival curves for the expanded efficacy studies (n=10 mice/condition) for each of the six EWS PDX models (SJ18 **(A),** SJ49 **(B)**, SJ17 **(C)**, NCH1 **(D)**, NCH4 **(E)**, and NCI21 **(F)**). Curves reflect survival to tumor size endpoint. Log-rank (Mantel-Cox) test was performed for each model to compare the treated groups to the vehicle. (* indicates p≤0.05, ** indicates p≤0.01, *** indicates p≤0.001, **** indicates p≤0.0001). Additional Log-rank (Mantel-Cox) test was performed to compare Irinotecan to LMP400 for each model. p-values are as follows: SJ18 **(A)**: p=0.0512, SJ49 **(B)**: p=0.0732, SJ17 **(C)**: p=0.7905, NCH1 **(D)**: p<0.0001, NCH4 **(E)**: p<0.0001, NCI21 **(F)**: p<0.0001.
