# Supplementary material for: Treatment with novel topoisomerase inhibitors in Ewing sarcoma models reveals heterogeneity of tumor response
Source: Front Cell Dev Biol. 2024 Oct 24;12:1462840. doi: 10.3389/fcell.2024.1462840 (PMC11542432; doi:10.3389/fcell.2024.1462840)
Supplement: Supplementary file 7 [file Image8.pdf]

Supplemental Figure S8

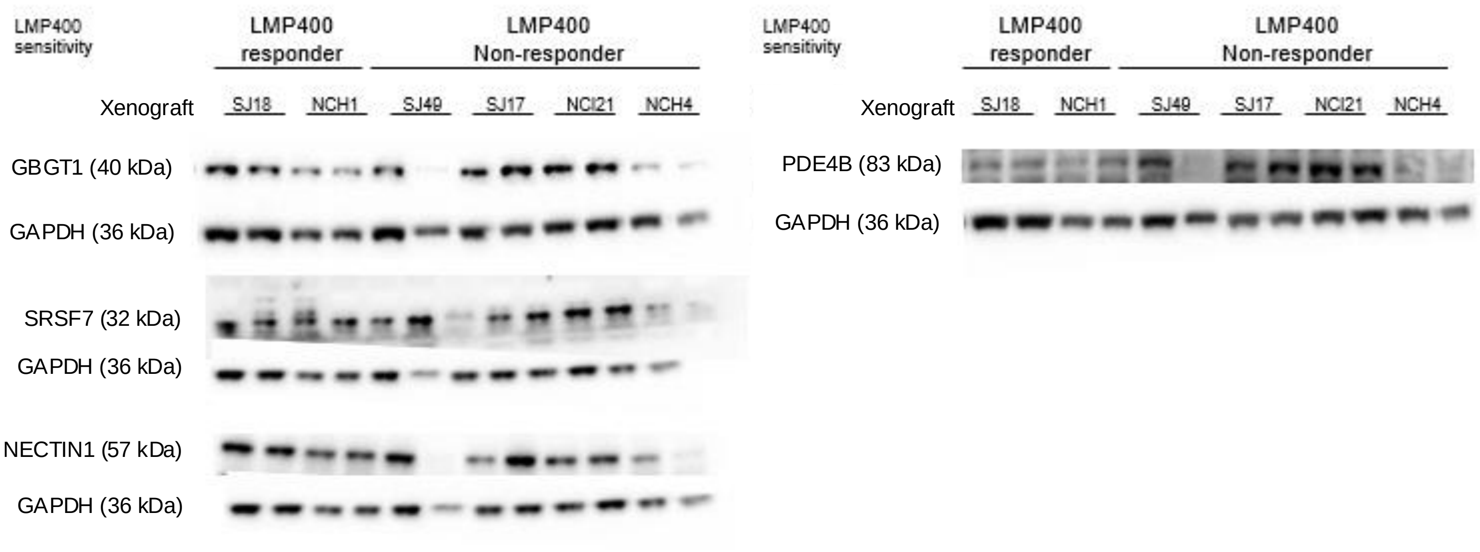

Supplemental Figure S8. Protein expression of four potential predictive biomarkers identified from the total RNA-seq are non-concordant with measured RNA expression levels. Western Blot showing the protein expression of GBGT1, SRSF8, NECTIN1, and PDE4B, genes for which protein expression was not concordant with RNA expression. At least two biological replicates for each PDX were included. TENM2 (shown in **Figure 4C**), SRSF8, and NECTIN1 were probed on the same membrane; ACSF2 (shown in **Figure 4C)**, GBGT1, and TSPAN8 (shown in **Figure 4C**) were probed on the same membrane, using the same loading controls.
