# Supplementary material for: Treatment with novel topoisomerase inhibitors in Ewing sarcoma models reveals heterogeneity of tumor response
Source: Front Cell Dev Biol. 2024 Oct 24;12:1462840. doi: 10.3389/fcell.2024.1462840 (PMC11542432; doi:10.3389/fcell.2024.1462840)
Supplement: Supplementary file 8 [file Image4.pdf]

Supplemental Figure S4

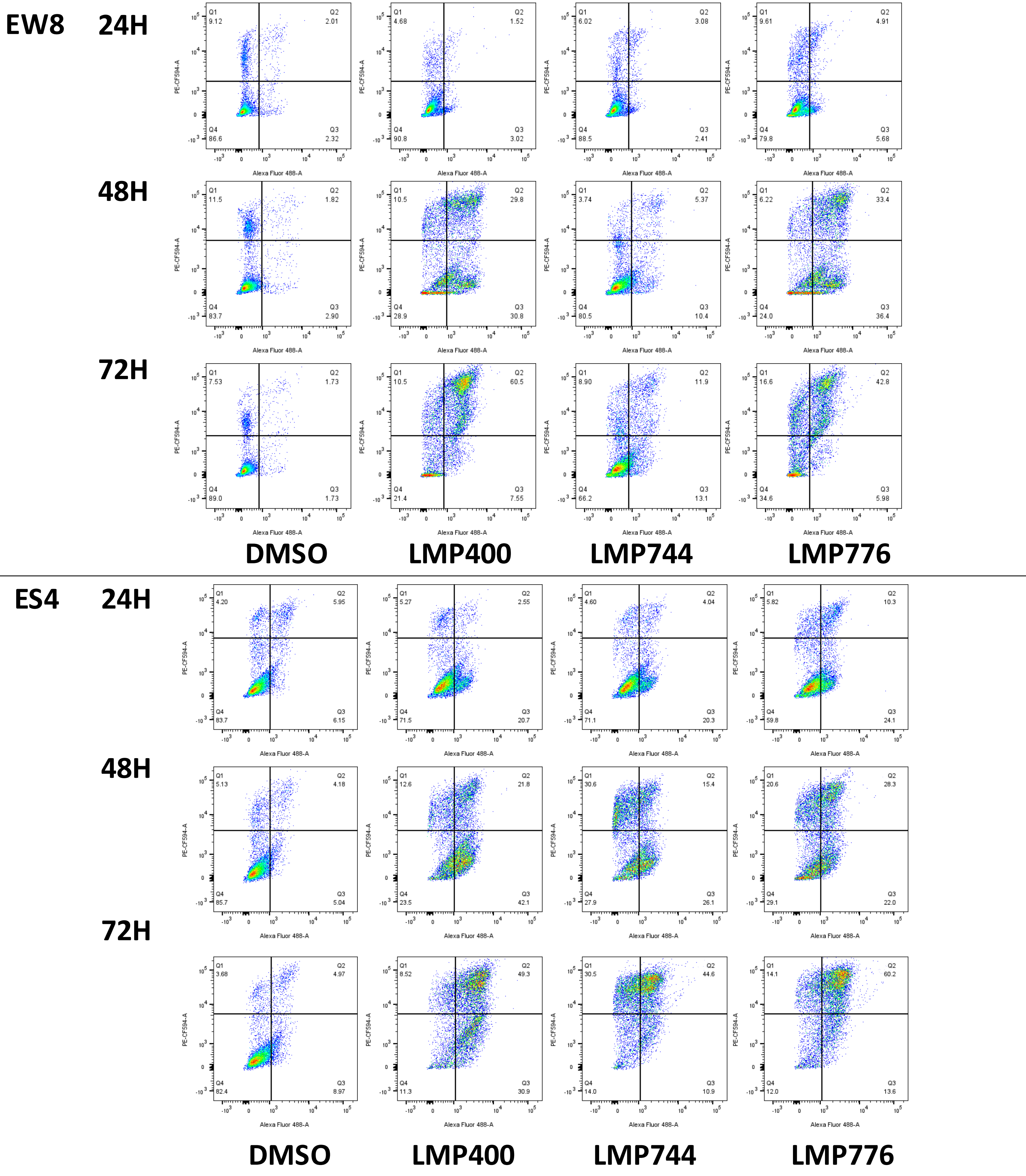

Supplemental Figure S4. IIQ treatments demonstrate time-dependent accumulation of early and late apoptotic populations in EWS cell lines. Representative flow plots as analyzed by FlowJo showing the shift towards early (Q3) and late (Q2) apoptosis of EW8 and ES4 cell lines treated for 24, 48, and 72 hours with DMSO, LMP400 (40 nM), LMP744 (80 nM for EW8 and 40 nM for ES4), or LMP776 (40 nM).
