# Supplementary material for: Treatment with novel topoisomerase inhibitors in Ewing sarcoma models reveals heterogeneity of tumor response
Source: Front Cell Dev Biol. 2024 Oct 24;12:1462840. doi: 10.3389/fcell.2024.1462840 (PMC11542432; doi:10.3389/fcell.2024.1462840)
Supplement: Supplementary file 9 [file Image2.pdf]

Supplemental Figure S2

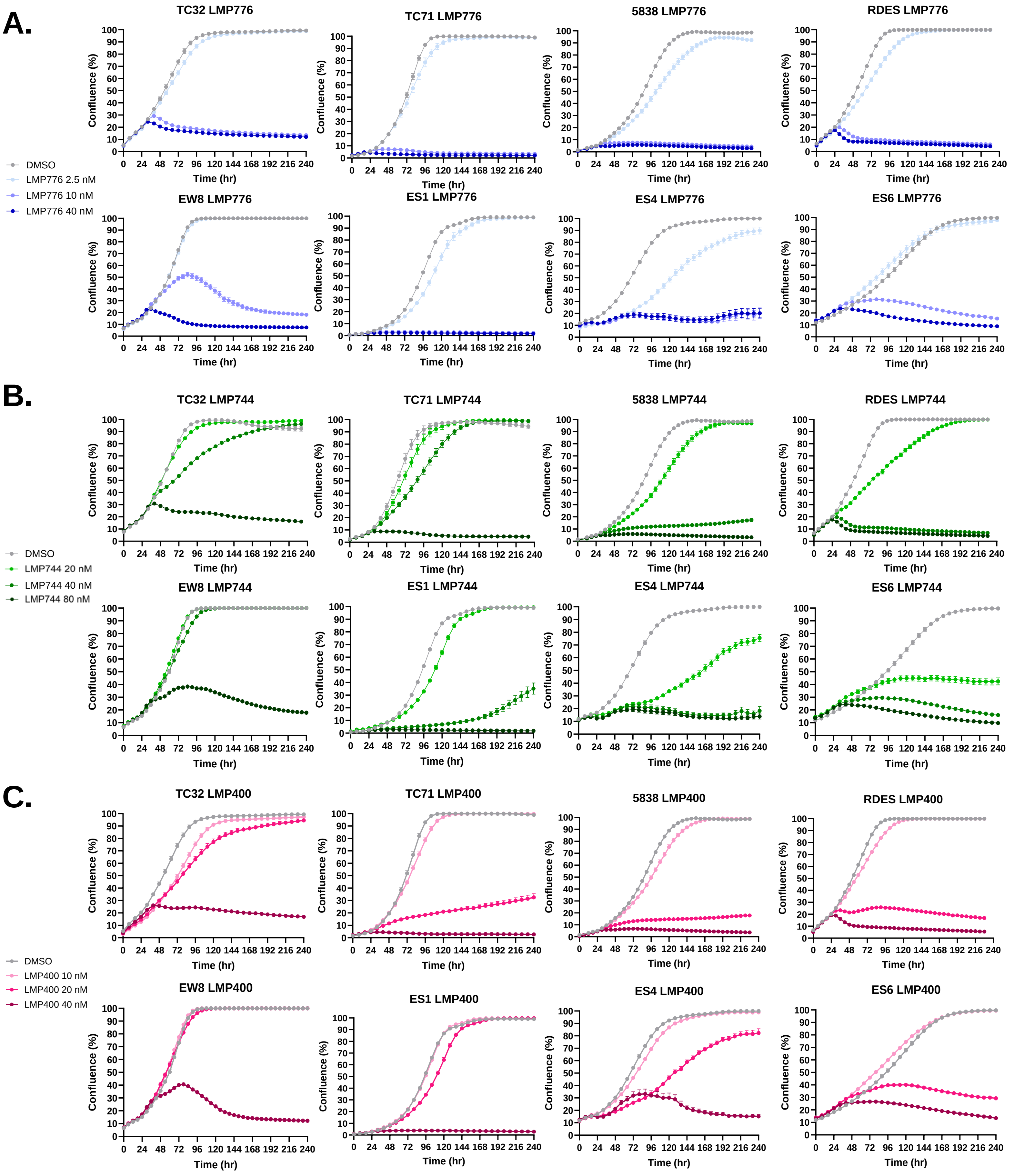

Supplemental Figure S2. IIQ treatments have dose-dependent anti-proliferative effects on EWS cell lines. IncuCyte live-cell analysis of eight EWS cell lines (TC32, TC71, 5838, RDES, EW8, ES1, ES4, ES6) treated with either DMSO (grey), LMP776 (**A.** shades of blue), LMP744 (**B.** shades of green), or LMP400 (**C.** shades of magenta), at three different doses.
