# Supplementary material for: Treatment with novel topoisomerase inhibitors in Ewing sarcoma models reveals heterogeneity of tumor response
Source: Front Cell Dev Biol. 2024 Oct 24;12:1462840. doi: 10.3389/fcell.2024.1462840 (PMC11542432; doi:10.3389/fcell.2024.1462840)
Supplement: Supplementary file 10 [file Image3.pdf]

Supplemental Figure S3

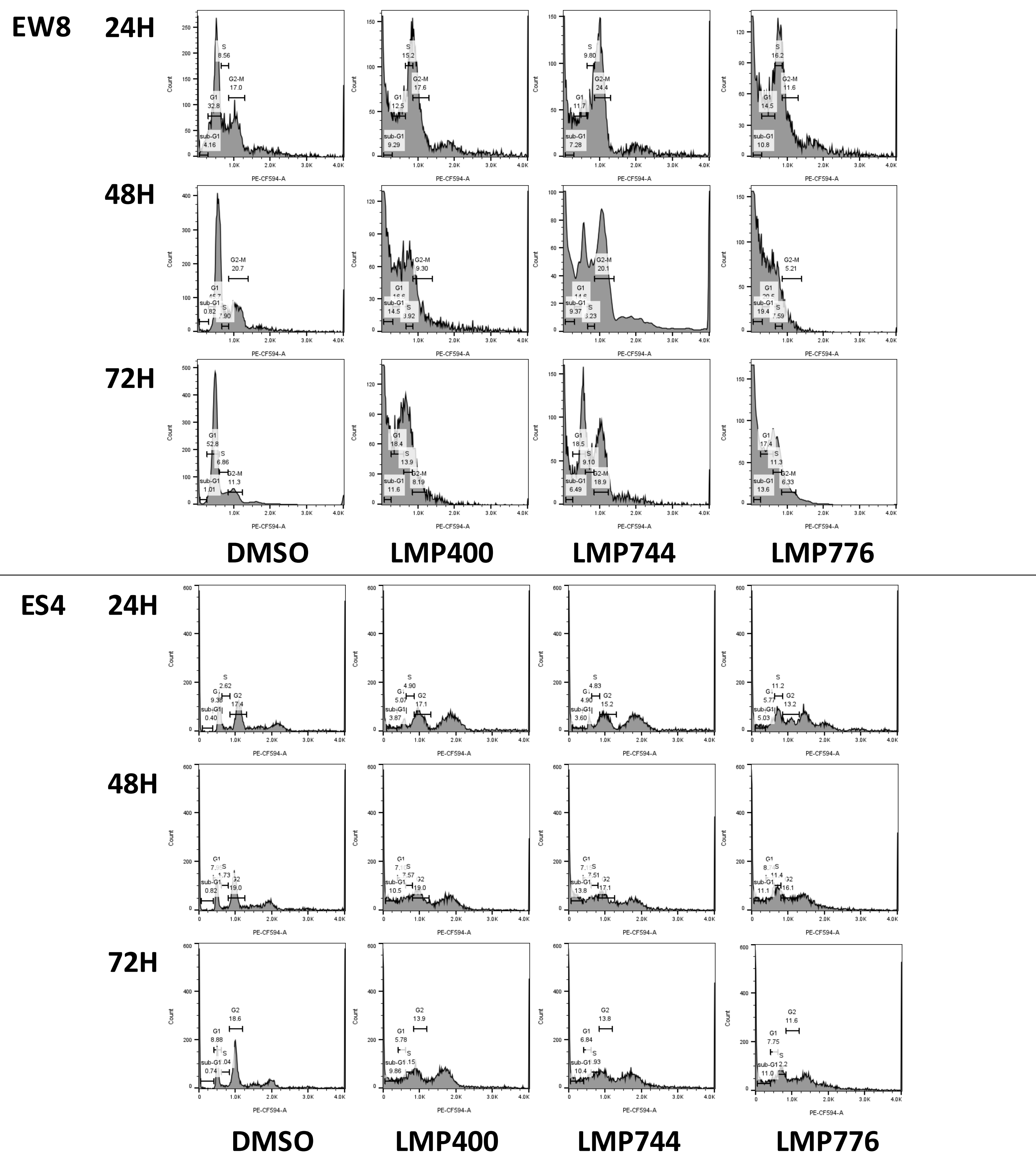

Supplemental Figure S3. IIQ treatments demonstrate time-dependent accumulation of sub-G1 population in EWS cell lines. Representative histograms as analyzed by FlowJo showing the changes in cell cycle of EW8 and ES4 cell lines treated for 24, 48, and 72 hours with DMSO, LMP400 (40 nM), LMP744 (80 nM for EW8 and 40 nM for ES4), or LMP776 (40 nM).
