# Supplementary material for: Treatment with novel topoisomerase inhibitors in Ewing sarcoma models reveals heterogeneity of tumor response
Source: Front Cell Dev Biol. 2024 Oct 24;12:1462840. doi: 10.3389/fcell.2024.1462840 (PMC11542432; doi:10.3389/fcell.2024.1462840)
Supplement: Supplementary file 11 [file Table3.pdf]

Treatment with novel topoisomerase inhibitors in Ewing sarcoma models reveals heterogeneity of tumor response

Unsun Lee, Ludmila Szabova, Victor J. Collins, Melanie Gordon, Kristine Johnson, Deborah Householder, Stephanie Jorgensen, Lucy Lu, Laura Bassel, Fathi Elloumi, Cody J. Peer, Ariana E. Nelson, Sophia Varriano, Sudhir Varma, Ryan D. Roberts, Zoe Weaver Ohler, William D. Figg, Shyam K. Sharan, Yves Pommier, Christine M. Heske

Supplemental Table S3. Details of tolerability study of 5-day-on/9-day-off/5-day-on schedule.

Compound	Dose	Route	Schedule	N
LMP400	15 mg/kg	IV	5-day-on/9-day-off/5-day-on	5
	20 mg/kg	IV	5-day-on/9-day-off/5-day-on	5
LMP744	10 mg/kg	IV	5-day-on/9-day-off/5-day-on	5
	15 mg/kg	IV	5-day-on/9-day-off/5-day-on	5
LMP776	5 mg/kg	IV	5-day-on/9-day-off/5-day-on	5
	10 mg/kg	IV	5-day-on/9-day-off/5-day-on	5
