# Supplementary material for: Treatment with novel topoisomerase inhibitors in Ewing sarcoma models reveals heterogeneity of tumor response
Source: Front Cell Dev Biol. 2024 Oct 24;12:1462840. doi: 10.3389/fcell.2024.1462840 (PMC11542432; doi:10.3389/fcell.2024.1462840)
Supplement: Supplementary file 12 [file DataSheet1.pdf]

Treatment with novel topoisomerase inhibitors in Ewing sarcoma models reveals heterogeneity of tumor response

Unsun Lee, Ludmila Szabova, Victor J. Collins, Melanie Gordon, Kristine Johnson, Deborah Householder, Stephanie Jorgensen, Lucy Lu, Laura Bassel, Fathi Elloumi, Cody J. Peer, Ariana E. Nelson, Sophia Varriano, Sudhir Varma, Ryan D. Roberts, Zoe Weaver Ohler, William D. Figg, Shyam K. Sharan, Yves Pommier, Christine M. Heske

Supplemental Methods

Histopathological Analysis

Tissues were fixed in 10% neutral buffered formalin and processed for paraffin embedding, sectioning and H&E staining. Histopathological examination of tumors was performed by a board-certified veterinary pathologist (L.B.) and indicated consistency with Ewing sarcoma histology. Ewing sarcoma histology was additionally confirmed by immunohistological staining for marker CD99.

Pharmacokinetic Analysis

Drug-free mouse plasma (BALB/c, NaHep, pooled gender) was purchased from BioreclamationIVT® (Baltimore, MD) and stored at −80 ℃. Optima® HPLC-grade acetonitrile was purchased from Fisher Scientific (Pittsburgh, PA), and dimethyl sulfoxide (DMSO) was purchased from Sigma-Aldrich (St. Louis, MO). Water used for the preparation of mobile phases and stock solutions was deionized and ultra-filtered on a MilliPore system (EMD MilliPore, Billerica, MA).

To examine the PK profile of the IIQs following their IV or IP administration, mouse blood and tumors were collected at different time points. Blood was collected into EDTA coated tubes and processed for plasma separation. Plasma and collected tumor pieces were flash frozen in liquid nitrogen. Three mice were collected for each time point and drug administration route. In three separate mice, vehicle (1 part of 20 mM HCl/10 mM citric acid and 9 parts of 5% dextrose water) was administered IP or IV as a control and blood and tumor samples were harvested at 2 hours.

Master stock solutions of LMP400, LMP776, and LMP744 were prepared by adding 1 mL of DMSO to each mg of drug to achieve final drug concentrations of 1 mg/mL. A stock solution was prepared from the 1 mg/mL master stock solutions of LMP400, LMP776, and LMP744. All three master stocks were combined and diluted into DMSO to achieve final drug concentrations of 100,000 ng/mL. The resulting stock solution was vortexed and then serially diluted into DMSO to the following concentrations: 10,000, 5,000, 1,000, 500, 100, 50, 10, and 5 ng/mL. All stock solutions were transferred to amber Waters HPLC glass vials and stored at −80 ℃.

Plasma Sample Preparation

Blank mouse plasma (BALB/c, NaHep, pooled gender) was used as matrix for the preparation of calibration curves and quality control (QC) samples. All calibration standards were prepared daily in duplicate by diluting the stock solutions 10-fold into matrix, providing a final calibration range of 0.5–1,000 ng/mL and 10% DMSO (v/v). Concentrations for low, mid, and high QC samples, prepared daily in quintuplet, were 1.5, 400, and 800 ng/mL, respectively. A lower limit of quantitation quality control (LLQC) of 0.5 ng/mL was also prepared daily in quintuplet for the validation. To account for study plasma samples exceeding the calibration range, a 10-fold dilution quality control (DCQ) of 10,000 ng/mL

was validated and included as necessary. In all QC samples, final DMSO content was held constant at 10% (v/v).

To 20 μL of calibration, QC, and study sample plasma, 250 μL of acetonitrile was added for protein precipitation. Samples were vortexed and centrifuged for 10 minutes at 4 ℃ and 13,300 RPM to separate the organic and aqueous layers. Exactly 220 μL of the supernatant was then transferred to a 96-well 2 mL plate, which was subsequently centrifuged for 10 minutes at 4 ℃ and 3,000 RPM.

Assay calibration for LMP400, LMP776, and LMP744 was achieved on an 8-point curve (0.50–1,000 ng/mL) and least-squares quadratic regression by plotting the analyte peak area versus the analyte concentration (ng/mL). For each calibration curve, a weighing factor of 1/x was implemented, where x is the concentration of analyte.

Tissue Sample Preparation

All calibration standards and quality control (QC) samples were prepared from the 100,000 ng/mL LMP400, LMP776, and LMP744 stock solutions. To prepare blank homogenate, 10 μL of water was first added to each mg of blank mouse tissue to achieve a final tissue concentration of 100 mg/mL. The tissue was then homogenized for a minimum of 1 minute with a Fisher Scientific™ PowerGen™ 125 and was used immediately for sample preparation.

Blank mouse homogenate was used as matrix for the preparation of calibration curves and quality control (QC) samples. Calibration standards were prepared daily in duplicate by serially diluting the stock solution into blank homogenate to the following concentrations: 20,000, 5,000, 2,500, 500, 50, 10, and 5 pg/mg. Similarly, QC samples were prepared via serial dilution of the stock solution into blank homogenate to the following concentrations: 15,000 (high), 3,000 (mid), and 15 pg/mg (low). A lower limit of quantitation quality control (LLQC) of 5 pg/mg was also prepared daily in quintuplet for the validation. Study samples of tissue were independently weighed, where 10 μL of water added per mg sample weight (100 mg/mL), then transferred to a BeadBug® tube containing 1.0mm high impact zirconium beads and homogenized on a Benchmark Scientific (Edison, NJ) BeadBlaster™ 24 using for 90 seconds.

To 50 μL of calibration, QC, and study sample homogenate, 500 μL of acetonitrile was added for protein precipitation. Samples were then vortexed and centrifuged for 10 minutes at 4 ℃ and 13,300 RPM to separate the organic and aqueous layers. Exactly 250 μL of the supernatant was transferred to a 96-well 2 mL plate, which was subsequently centrifuged for 10 minutes at 4 ℃ and 3,000 RPM.

Assay calibration for LMP400, LMP776, and LMP744 was achieved on a 7-point curve (5 – 20,000 pg/mg) and least-squares quadratic regression by plotting the analyte peak area versus the analyte concentration (ng/mL). For each calibration curve, a weighing factor of $1/x^2$ was implemented, where x is the concentration of analyte.

Instrumentation and Chromatography

A Waters ACQUITY UPLC® (Waters Corporation, Milford, MA, USA) with a binary solvent pump, temperature-controlled column compartment (35 ℃) and refrigerated autosampler (4 ℃) was fitted with a Waters ACQUITY UPLC® BEH C18, 2.1×50 mm, 1.7 μm column. A total volume of 10 μL of sample was injected into the column. Mobile phases were 0.1% formic acid (aq) and 0.1% formic acid in acetonitrile. The following gradient was established over the course of a 4-minute run at a flow rate of

0.40 mL/minute: 10% organic (initial), 80% organic (1.6 minutes), 10% organic (2.6 minutes to end). An AB Sciex QTRAP 5500 mass spectrometer (AB Sciex, Framingham, MA) was used to monitor transitions of LMP400 (*m/z* 479.2→392.4), LMP744 (*m/z* 460.1→392.1), and LMP776 (*m/z* 453.2→392.2) using multiple reaction monitoring (MRM) in the positive ion mode. Collision energies for LMP400, LMP744, and LMP776 were 40V, 25V, and 30V, respectively. Source parameters used for all analytes were a declustering potential (DP) of 20V, curtain gas of 30 psi, collision gas (CAD) of medium, ion source temperature (TEM) of 500 ℃, ionspray voltage (IS) of −5500V, nebulizing gas (GS1) of 40 psi, drying gas (GS2) of 40 psi, entrance potential of −10V, and an exit potential of −13V.
