# Supplementary material for: Treatment with novel topoisomerase inhibitors in Ewing sarcoma models reveals heterogeneity of tumor response
Source: Front Cell Dev Biol. 2024 Oct 24;12:1462840. doi: 10.3389/fcell.2024.1462840 (PMC11542432; doi:10.3389/fcell.2024.1462840)
Supplement: Supplementary file 13 [file Image7.pdf]

Supplemental Figure S7

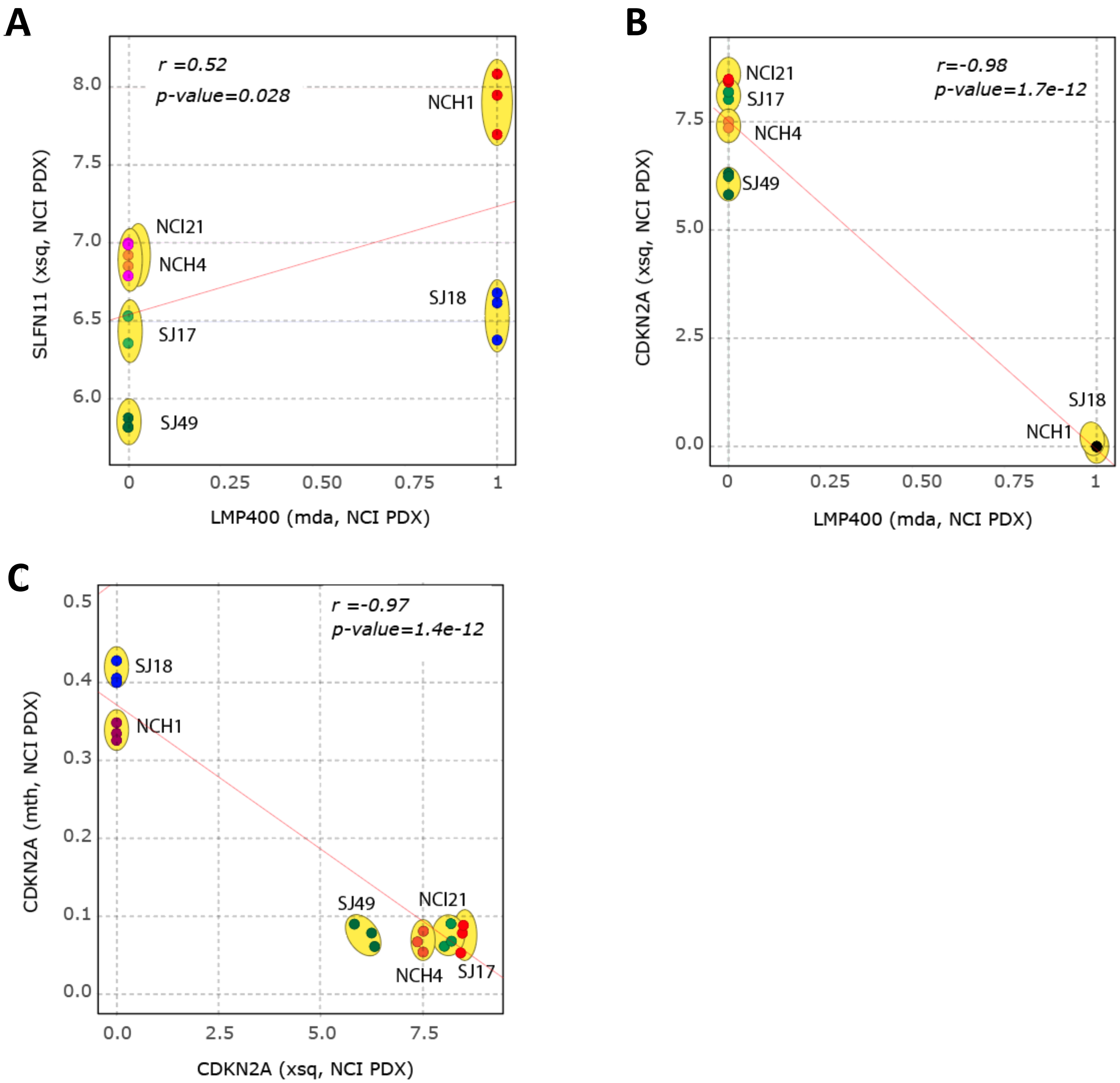

Supplemental Figure S7. Representative snapshots of Ewing Sarcoma MinerCDB (https://discover.nci.nih.gov/rsconnect/EwingSarcomaMinerCDB) **showing data reproducibility across PDX and insights into SLFN11 and CDKN2A gene expression and LMP400 response. A.** Expression of *SLFN11* as a function of LMP400 response. **B.** Expression of *CDKN2A* as a function of LMP400 response (1 and 0 denote >30% and <30% response). **C.** Epigenetic inactivation of CDKN2A in the NCH1 and SJ18 PDXs. Clustering of the PDX triplicates is indicated as yellow elliptical highlights. Statistical analyses (Pearson correlation coefficients and p-values are annotated with the regression lines shown in red).
