# Supplementary material for: Treatment with novel topoisomerase inhibitors in Ewing sarcoma models reveals heterogeneity of tumor response
Source: Front Cell Dev Biol. 2024 Oct 24;12:1462840. doi: 10.3389/fcell.2024.1462840 (PMC11542432; doi:10.3389/fcell.2024.1462840)
Supplement: Supplementary file 14 [file Image1.pdf]

Supplemental Figure S1

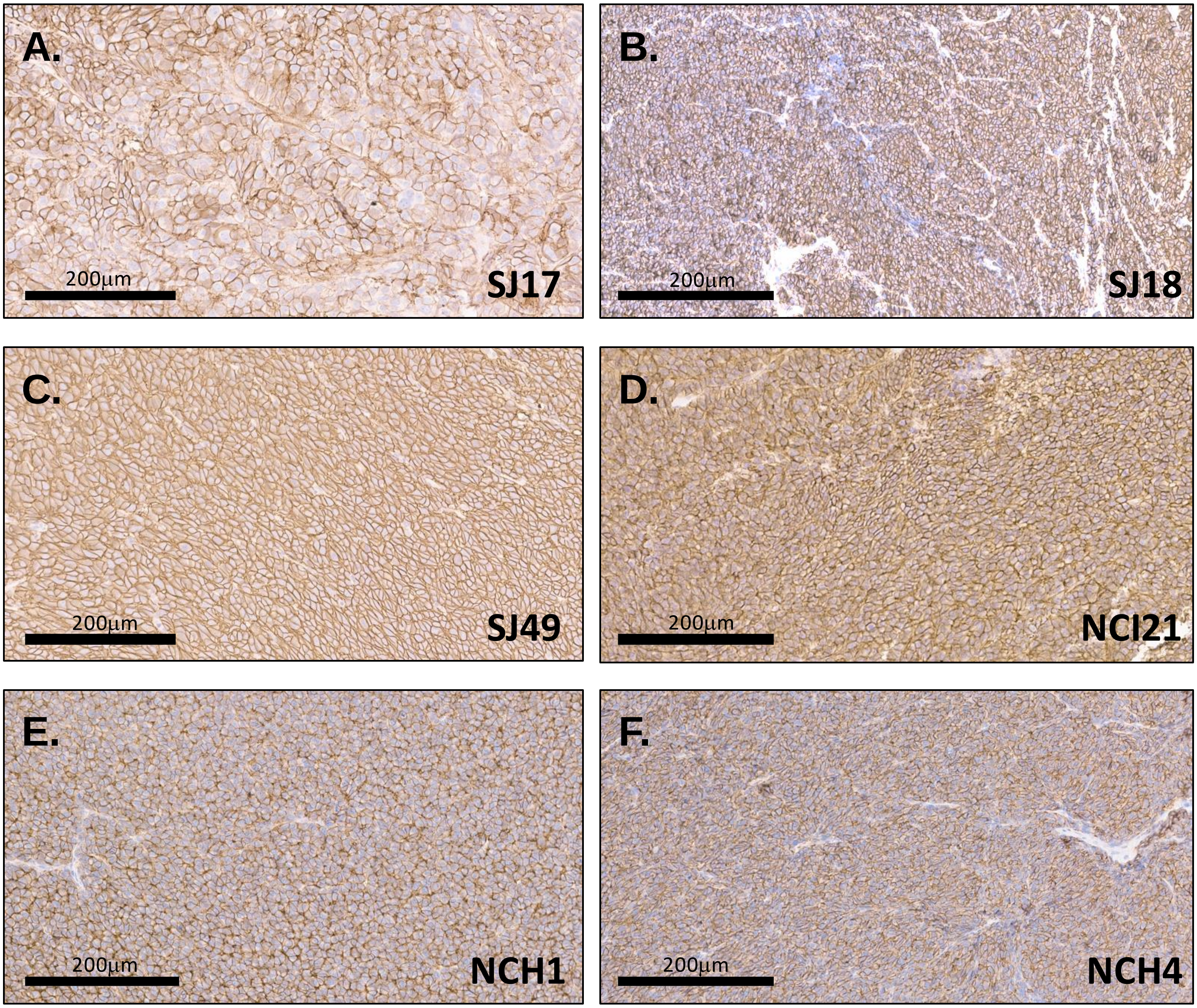

Supplemental Figure S1. PDX models of EWS are positive for CD99 marker. IHC results confirming positive membranous staining (brown) for CD99 marker in all examined PDX models: **A.** SJ17, **B.** SJ18, **C.** SJ49, **D.** NCI21, **E.** NCH1, **F.** NCH4.
